# Head and neck tumor organoid biobank for modelling individual responses to radiation therapy according to the TP53/HPV status

**DOI:** 10.1186/s13046-025-03345-3

**Published:** 2025-03-05

**Authors:** Christian Issing, Constantin Menche, Mara Romero Richter, Mohammed H. Mosa, Jens von der Grün, Maximilian Fleischmann, Philipp Thoenissen, Ria Winkelmann, Tahmineh Darvishi, Andreas G. Loth, Shahram Ghanaati, Franz Rödel, Peter J. Wild, Christian H. Brandts, Timo Stöver, Henner F. Farin

**Affiliations:** 1 Department of Otorhinolaryngology, Goethe University Frankfurt, University Hospital, Frankfurt/Main, Germany; 2 Georg-Speyer-Haus, Institute for Tumor Biology and Experimental Therapy, Frankfurt/Main, Germany; 3 Frankfurt Cancer Institute, Goethe University Frankfurt, Frankfurt/Main, Germany; 4 University Cancer Center (UCT) Frankfurt, Goethe University Frankfurt, University Hospital, Frankfurt/Main, Germany; 5 Mildred-Scheel Early Career Center Frankfurt, Frankfurt/Main, Germany; 6 German Cancer Consortium (DKTK), Frankfurt/Mainz partner site and German Cancer Research Center (DKFZ), Heidelberg, Germany; 7 Department of Radio-oncology, University Hospital Zürich, Zürich, Switzerland; 8 Department of Radiotherapy and Oncology, Goethe University Frankfurt, University Hospital, Frankfurt/Main, Germany; 9 Clinic of Oral, Cranio-Maxillofacial and Plastic Facial Surgery, Goethe University Frankfurt, University Hospital, Frankfurt/Main, Germany; 10 Dr. Senckenberg Institute for Pathology and Human Genetics, Goethe University Frankfurt, University Hospital, Frankfurt/Main, Germany; 11 Department of Medicine, Hematology/Oncology, Goethe University Frankfurt, University Hospital, Frankfurt/Main, Germany

**Keywords:** Head and neck squamous cell carcinoma, Tumor organoid biobank, Preclinical model, In vitro therapy testing, Radioresistance, Genetic editing, CRISPR/Cas9

## Abstract

**Background:**

Head and neck cancers (HNC) represent an extremely heterogeneous group of diseases with a poorly predictable therapy outcome. Patient-derived tumor organoids (PDTO) offer enormous potential for individualized therapy testing and a better mechanistic understanding of the main HNC drivers.

**Methods:**

Here, we have established a comprehensive molecularly and functionally characterized head and neck organoid biobank (HNOB) recapitulating the clinically relevant subtypes of *TP53* mutant and human papillomavirus type 16 (HPV 16) infection-driven HNC. Organoids were exposed to radiotherapy, and responses were correlated with clinical data. Genetically engineered normal and tumor organoids were used for testing the direct functional consequences of TP53-loss and HPV infection.

**Results:**

The HNOB consisting of 18 organoid models, including 15 tumor models, was generated. We identified subtype-associated transcriptomic signatures and pathological features, including sensitivity to TP53 stabilization by the MDM2 inhibitor Nutlin-3. Furthermore, we describe an in vitro radio response assay revealing phenotypic heterogeneity linked to the individual patient’s treatment outcome, including relapse probability. Using genetically engineered organoids, the possibility of co-existence of both cancer drivers was confirmed. *TP53* loss, as well as HPV, increased growth in normal and tumor organoids. *TP53* loss-of-function alone was insufficient to promote radiation resistance, whereas HPV 16 oncogenes E6/E7 mediated radiosensitivity via induction of cell cycle arrest.

**Conclusion:**

Our results highlight the translational value of the head and neck organoid models not only for patient stratification but also for mechanistic validation of therapy responsiveness of specific cancer drivers.

**Supplementary Information:**

The online version contains supplementary material available at 10.1186/s13046-025-03345-3.

## Background

Head and neck cancers (HNC) represent a highly heterogeneous group of upper aerodigestive tract malignancies, including the oral cavity, pharynx, and larynx [1]. The most common type of cancer in this region are head and neck squamous cell carcinoma (HNSCC), originating from the stratified epithelium [1–3]. While the epithelium provides a protective barrier, it is also potentially exposed to genotoxic agents like alcohol or tobacco-derived carcinogens. Common genetic alterations are loss of function mutations of the tumor suppressor protein 53 gene (*TP53*) and less frequently mutations in *CDKN2A*, *TTN*, *FAT1*, or *PIK3CA* [1, 4–7]. On the other hand, the incidence of human papillomavirus (HPV)-associated HNC continues to rise, with HPV 16 as the most prevalent subtype, mainly driven by the viral oncogenes E6 and E7 [8–12]. Unfortunately, our increased knowledge about the molecular alterations of HNC has not yet led to a more personalized therapy, and most identified biomarkers have not been successfully integrated into clinical routine [6, 13–17]. At present, the distinction between HPV-positive (+) and HPV-negative (–) tumors is most relevant. p16-staining can serve as an HPV surrogate marker and has been included in the latest TNM classification [18], although its reliability as a biomarker has been questioned in the literature [19–22]. Importantly, HPV + oropharyngeal squamous cell carcinoma associated with a more favorable prognosis, which has led to radiation dose de-escalation strategies in recent clinical trials [22–24].

The treatment of localized HNSCC includes surgical resection, and if applicable, combined with (neo-)adjuvant radio(chemo)therapy. At locally advanced stages, the resection is often associated with significant functional impairment representing a particular clinical challenge. As an alternative to surgery, definitive radiotherapy combined with cisplatin-based chemotherapy can be performed with comparable overall survival. Chemoradiotherapy is the treatment of choice for inoperable stages [1, 9, 25, 26]. Further, checkpoint inhibitors targeting PD-1 and PD-L1 are becoming increasingly important with a positive effect on overall survival in recurrent or metastatic settings [27–29]. However, five-year survival today in advanced HNSCC is only about 50%, and in recurrent situations, the average survival is about one year [1, 27]. Together, there is an urgent need for an improved mechanistic understanding of HNSCC and a better prediction of the individual therapy response.

Patient-derived tumor organoids have been established as a preclinical in vitro tool for therapy testing in colorectal cancer and other tumor entities [30–36]. Recently, culture conditions for HNSCC organoids were published that accurately preserved the patient-specific molecular and phenotypic tumor characteristics [37]. PDTO biobanks can serve as a valuable resource to study personalized responses to treatments, validate potential biomarkers and model disease progression by targeted genetic modifications [30, 31, 34, 37–44]. Here, we have established the *head and neck organoid biobank (HNOB)* to address two key aspects: first, to characterize individual and cancer driver-specific tumor characteristics and second, to generate genetically defined *TP53*/HPV models to address driver-specific consequences on the tumor phenotype and response to radiation therapy.

## Methods

### Collection of clinical samples and data

Patients with histologically confirmed HNC were recruited at the Department of Oto-Rhino-laryngology and the Department of Oral, Maxillofacial and Facial Plastic Surgery, Goethe University Frankfurt, between 12/2020 and 10/2022. Written informed consent was obtained from all patients. All materials were collected via the interdisciplinary biobank of the University Cancer Center Frankfurt (UCT). The study was approved by the “Scientific Board” of the interdisciplinary Biobank and Database Frankfurt (iBDF) and the ethics committee at the University Cancer Center Frankfurt (project number: UCT-6-2020). 18 resections (15 tumors and 3 adjacent normal tissues) were collected from 16 different patients. Samples were pseudonymized and linked to clinical data (Supplementary Table 1). Simultaneously, routine pathological examination of formalin-fixed paraffin-embedded specimens was performed. Fresh tissue samples were rapidly stored in cold washing medium (DMEM [Thermo Fisher Scientific], 0,4% BSA [Sigma-Aldrich] and 1x penicillin/streptomycin [Thermo Fisher Scientific]) on ice. Necrotic/ burned areas were removed, and the tissue was dissected with a scalpel. Several representative pieces were selected and frozen from each sample at -80 °C for DNA isolation.

### Tissue processing for organoid generation

Organoid cultures were established and maintained as described [37] with adaptations to improve long-term cultivation. Briefly, tissue pieces were collected, washed in an ice-cold washing medium and PBS, followed by incubation at 37 °C in 0.125% Trypsin (Sigma-Aldrich). Digestion was visually inspected, stopped by the addition of washing medium, filtrated (100 μm EasyStrainer, Greiner), and centrifugated. The cell pellet was washed in a cold washing medium, centrifuged, and resuspended in 75% Growth factor-reduced BME type 2 (Cultrex). BME domes, approximately 10 μl each were seeded, followed by addition of culture medium containing: advanced DMEM/F12 supplemented with 10 mM HEPES, 1× Glutamax, 1× penicillin/streptomycin, 2% B27 (Life Technologies), 12.5 mM N-acetylcysteine (Sigma-Aldrich), 500 nM A83-01 (R&D Systems), 50 ng/ml human EGF (PeproTech), 25 ng/mL human FGF2 (PeproTech), 10 ng/mL human FGF10 (PeproTech), 1 μmol/L Prostaglandin E2 (Tocris Bioscience), 1 μmol/L Forskolin (Bio-Techne), 20% R-spondin 1 conditioned medium, 20% Wnt/Afamin conditioned medium and 10% Noggin conditioned medium. Conditioned media (CM) were prepared as described (Farin et al., 2012) [45]. Wnt/Afamin CM was prepared using EXPI 293 cells as described (Mihara et al., 2016) [46] in the absence of serum and stored at -20 °C before use. After seeding and splitting, 10 μM of Y-27,632 was added to the medium for the first 3 days. 100 μg/ml Primocin (InvivoGen) was added for the first three passages. The medium was changed twice per week, and organoids were split every 7–14 days at a ratio of 1:2 to 1:4 by enzymatic digestion with StemPro Accutase (Gibco) at 37 °C. Digestion was regularly checked, and the suspension was filtered (70 μm EasyStrainer, Greiner) to exclude remaining organoid fragments. Master aliquots for each organoid line were cryopreserved at low passage numbers, and all PDOs were kept in culture for a limited number of passages and then replaced with new master aliquots.

### DNA/RNA isolation of organoids

For DNA isolation, organoids were collected 7 days after seeding. DNA extraction was performed with the DNeasy Blood & Tissue Kit (Qiagen following the manufacturer´s suggestions. In parallel, cryopreserved tumor tissue pieces were processed and DNAs were eluted in 100 μl PCR-grade water, measured using Invitrogen Qubit 2.0 Fluorometer (ThermoFisher Scientific, Waltham, MA, USA) and stored at -80 °C before analysis. For RNA isolation, organoids were seeded in the colony formation assay condition (see below) and collected 2 or 7 days after seeding. RNA extraction was performed with the NucleoSpin RNA set (Machery-Nagel) following the manufacturer´s suggestions. RNA was eluted in 50 μl RNase-free water, measured using NanoDrop and stored at -80 °C before analysis.

### Whole exome sequencing (WES) analysis

Genomic DNA from the PDTO and ten matched tumor tissues were subjected to whole exome analysis at Azenta (Leipzig, Germany) as follows: Library preparation was performed using Agilent SureSelect Human All Exon V6 chemistry according to Azenta. Briefly, genomic DNA was fragmentated with a Covaris S220, cleaned up, end repaired and adenylated at the 3’ends. Next, DNA fragments were ligated to adapters. For validation Agilent 5300 Fragment Analyzer (Agilent Technologies, Palo Alto, CA, USA), and for quantification Qubit 4.0 Fluorometer (Invitrogen, Carlsbad, CA) were used. Then, adapter-ligated DNA fragments were enriched with limited cycle PCR and hybridized with biotinylated baits. Using streptavidin-coated binding beads, the hybrid DNA was captured, extensively washed, amplified and indexed with Illumina indexing primers. Validation of the post-captured DNA libraries were performed by using Agilent 5300 Fragment Analyzer (Agilent Technologies, Palo Alto, CA, USA) and quantified using Qubit 4.0 Fluorometer (Invitrogen, Carlsbad, CA). After multiplexing, the libraries were loaded on the flow cell on the Illumina NovaSeq 6000 instrument according to the manufacturer’s suggestions. For sequencing, a 2 × 150 paired-end (PE) configuration v1.5 was used. Image analysis and base calling were conducted by the NovaSeq Control Software v1.7 on the NovaSeq instrument. Files were converted into Fastq files and de-multiplexed using Illumina bcl2fastq program version 2.20. One mismatch was allowed for index sequence identification. Data Analysis: Variant calling was performed as published [47]. In brief, sequencing adapters and low-quality bases in raw reads were trimmed using Trim Galore (version 0.6.10). Cleaned reads were then aligned to the GRCh38 reference genome using bwa-mem2 (version 2.2.1). Alignments were then sorted, and PCR/optical duplicates were marked by samtools (version 1.19.2). Somatic SNVs and INDELs were called by HaplotypeCaller (GATK, version 4.5.0.0), FreeBayes (version 1.3.6) and DeepVariant (version 1.6.0). The generated VCF files were then normalized (left alignment of INDELs and splitting multiallelic sites into multiple sites) and consolidated (variants called by all three tools) using bcftools (version 1.19). Consolidated VCF files were annotated and converted to MAF format using Ensembl Variant Effect Predictor (VEP; version v110).

Somatic variants were analyzed and visualized using maftools R package (version 2.18.0) [48]. Briefly, variants with less than 5% variant allele frequency (VAF) within the sample and variants with incidence > 0.01% in the gnomAD and 1k genomes databases were excluded from subsequent analysis. “plotmafSummary” was then used to plot mutation variant classification, variant type, single nucleotide variants (SNV) class using default parameters. Mutated genes of the TCGA Firehose cohort [49] were sorted according to the mutation frequency. Average mutation frequencies together with recurrent mutations were calculated using “oncoplot” function, considering only genes that had at least one mutation within the biobank cohort. TP53 variant mutations were visualized using “lollipopPlot” function. Mutant-allele tumor heterogeneity score as well as number of clones were inferred from the variant allele frequencies (VAF) using “inferHeterogeneity” function. Mutational concordance was calculated as following: a variant was deemed shared between the organoids and the original tumor if its VAF > 5% in both conditions. For cancer driver mutations, the filter was set to potentially relevant variants (using default maftools filters).

### RNA sequencing analysis

Eluted RNA was sent for sequencing at Genome Scan NL (Leiden, Netherlands). The sample library was prepared with NEBNext® Ultra II Direction RNA Library Prep Kit for Illumina (New England BioLabs, #E7760). The RNA was sequenced with the Illumina NovaSeq 6000 (PE 150). An average of 24 ± 4 (SD) million reads per sample were measured. The quality of Fastq files was checked using FastQC software (version 0.11.8). Fastq files were aligned to reference genome GRCh38 and aligned reads were assigned to genes using “Rsubread” (version 2.14.2) and Ensembl annotations (version 110). Gene annotation conversion was performed using the “AnnotationDbi” (version 1.62.2) and “org.Hs.eg.db” (version 3.17.0) packages. Differential gene expression was performed using “DESeq2” (version 1.40.2). Comparison between PDTOs was performed in unpaired (Supplementary Table 4a, 5a), between timepoints in a paired manner (Supplementary Table S4b, S5b). For plotting, readcounts of the top 100 genes were normalized using the “vst” function of DESeq2. Hierarchical clustering and gene heatmaps were plotted using “pheatmap” (version 1.0.12). For gene set enrichment analysis (GSEA), genes with base mean > 20 were ranked according to their log2 fold change. This list was compared to the MSigDB database (version 7.5.1) using “fgsea” (version 1.26.0) with minimum and maximum sizes of 10 and 2500 respectively (Supplementary Tables S4b, S5b). Normalized enrichment scores (NES) and adjusted *p*-values were plotted.

### Histology and immunhistochemistry

Organoids were harvested, fixed in 4% formalin for 45 min and embedded in 30 μl HistoGel (Thermo Scientific) before transfer to paraffin and sectioning. p16 staining (clone JC8; Dako Omnis, Agilent Technologies, Santa Clara, California) and p53 staining (clone DO-7; Dako Omnis, Agilent Technologies, Santa Clara, California) were performed at the Dr. Senckenberg Institute for Pathology, Frankfurt using an automated staining system, according to manufacturer´s protocol. P53 expressing cells were quantified using Leica Aperio eSlide Manager software (version 12.5) (Supplementary Fig. S4).

### Sequencing-based HPV analysis

Fastq files from RNA or WES sequencing were aligned to HPV16 (NC_001526.4, annotations: GCF_000863945.3) and HPV33 (GenBank: M12732.1, annotations: GCA_003179955.1) reference genomes. Aligned reads were assigned to genes using “Rsubread”. Samples were considered positive when more than one HPV specific gene was detected/expressed.

### Amplicon-based HPV genotyping

DNA of PDTOs and matched tumor tissue (as above) was PCR amplified using biotinylated primer sequences specific for the L1 region of the human papilloma virus (HPV) using the VisionArray HPV PreCise Master Mix (ZytoVision GmbH) according to the manufacturer’s instructions (www.zytovision.com). Subsequently, hybridization was performed between specific complementary immobilized DNA capture sequences on a glass chip (VisionArray HPV Chip 1.0). Non-bound DNA was washed off, before chips were scanned (VisionArray Scan system) and analyzed (VisionArray MultiScan software).

### Colony formation assay

Single cells were filtered (40 μm EasyStrainer, Greiner), counted and seeded in triplicates at 500, 1000, 2000, and 5000 cells per 10 μl BME. After seven days, images were taken, and the number of organoids was determined using ImageJ software. Linear regression analysis was performed to calculate the number of organoids/ 2000 input single cells. To ensure stable growth for the assays in all organoid lines and optimal comparability of the different treatments, the number of single cells required to form 300 organoids was determined for each organoid line.

### Nutlin-3 tolerance assay

A normalized cell number was seeded in 10 μl BME as triplicates. Nutlin-3 was dissolved in DMSO, and DMSO content was normalized in all wells. Organoids were grown in full organoid medium in different Nutlin-3 concentrations (0.5; 2.5; 5; 10; 20 μM) and DMSO a control. Medium was replenished after four days. After 7 days of drug exposure cell viability was assessed using CellTiter-Glo assay (Promega), according to the manufacturer’s instructions. The luminescence was measured using SpectraMax iD5 (Molecular Devices). Individual values were excluded if BME drops/ cells were lost due to technical errors or if seeding was not uniform.

### Radiosensitivity assay

A normalized cell number to obtain 300 organoids was seeded in 10 μl BME on a 96-microtiter plate (Greiner, 655090). Per condition six wells were seeded. Two days after seeding, organoid viability was determined using a modified RealTime-Glo™ MT Cell Viability Assay (Promega). Both MT Cell Viability Substrate and NanoLuc® Enzyme were added under light-protection at a concentration of 2x to the full medium. The organoids were incubated for 75 min at 37 °C and the luminescence was measured using SpectraMax iD5. Plates were irradiated with 0; 2; 4; 6; 8 and 10 Gy using linear accelerator (Synergy FL, ELEKTA) with a 6 MV photon energy, a 100 cm focus-surface distance, and a dose rate of 6 Gy/min at the Department of Oncology and Radiotherapy, University Hospital Frankfurt. Cell viability was measured after radiation every 48 h up to 144 h using RealTime-Glo™ (Promega) and the medium was changed every 48 h. Growth rate normalization was performed using the online tool “GR metrics” (http://www.grcalculator.org) [50]. Individual values were excluded if BME drops/ cells were lost due to technical errors or if seeding was not uniform. Cell doubling was calculated using the RealTime Glo data as described by Żuławińska, J. (“Cell Doubling Time Calculator”. Available at: https://www.omnicalculator.com/biology/cell-doubling-time. Accessed: 10 July 2024).

### Cloning of *TP53* DN lentivirus

For lentiviral overexpression the open reading frame (ORF) of the dominant negative (DN) *TP53*^*R248Q*^ variant was cloned into pCDH-CMV_Nluc_P2A-copGFP-T2A-Puro (Addgene, #73037) (Supplementary Fig. 6). RNA was isolated from O04T and reverse transcribed by adding 0.25 μg oligo deoxythymidines (dTs) primer (Promega) to 0.5 μg RNA and filled up to 35 μl total volume with UltraPure DNase/RNase-Free Water (ThermoFisher Scientific) incubated at 75 °C for 5 min followed by 5 min on ice. After addition of 0.75 μl RNAsin (Promega), 15 μl 5x Buffer; 3.125 μl dNTPs (10mM each) and 0.75 μl MuLV reverse transcriptase (200–300 U/μl) (Roche) up to 40 μl total volume the reaction was incubated for one hour at 45 °C, followed by 15 min at 75 °C. The ORF was then PCR amplified using the primers ctagagctagggatccaccATGGAGGAGCCGCAGTCAGATC and ctccactgccgtcgacgcgGTCTGAGTCAGGCCCTTCTGTCTTGA (Supplementary Table S7) The product was gel purified and introduced into the vector cut by the restriction enzymes BamHI-HF (New England Biolabs) and SgrDI (ThermoFisher Scientific) with In-Fusion HD enzyme premix (Takara Bio) according to the manufacturer’s instruction and transformed into 50 μl Stellar competent E. coli (Takara Bio). Bacterial plasmid DNA was collected and the ORF was confirmed by Sanger sequencing.

### Lentiviral transduction

Lentivirus production and organoid transduction were performed using a modified protocol as described [51]. Briefly, organoids were dissolved into single cells and resuspended in 800 μl +++ medium containing 10 μM Y-27,632 and 10 μg/ml polybrene (Merck). Next, the cells were spinfected (1000 x g, 32 °C, 1 h) and incubated for one hour at 37 °C, 5% CO_2_, pelleted, seeded out in BME, and covered with HNSCC medium containing 10 μM Y-27,632 and 500 μg/ml Primocin. For *TP53* ko, cells were infected with LentiCas9-Blast (Addgene, #52962) and pU6-TP53-sgRNA-IRES-Puro (Supplementary Fig. S7a). For selection, 1 μg/ml Puromycin (InvivoGen) and/or 1 μg/ml Blasticidin (Santa Cruz Biotechnology) were used. *TP53* ko was confirmed after passage 4 using Sanger sequencing (Supplementary Fig. S7b). The indel rate was determined with ICE assay [52]. The lentiviral plasmids pWPI-HPV-E6E7-Puro and pWPI-Puro were described [53], and all lentiviruses are listed in Supplementary Table S6.

### Western blot (WB)

*TP53* ko and *TP53* DN organoids and the corresponding control groups were treated with 10 μM Nutlin-3 for 24 h 4 days after seeding. Organoids were collected and lysed with 70 μl RIPA buffer containing 5% 1 M Tris-HCl (Carl Roth), 1% Nonidet P-40 (Fluka); 0.5% sodium deoxycholate (Merck), 0.1% sodium dodecyl sulfate (SDS, Carl Roth), 3% 5 M sodium chloride (NaCl, AppliChem), 0.4% 0.5 M EDTA (Carl Roth) and 0.2% sodium fluoride (NaF, Merck) in water with 0,1% proteinase inhibitor cocktail (Merck) for 15 min on ice. The lysate was centrifuged at 20,000 x g for 15 min at RT. Protein concentration was measured by Bradford-assay (Bio-Rad, spectrometer: Thermo Scientific, Evolution 60). 30 μg protein lysate was loaded on a 15% SDSpage before transfer to a nitrocellulose membrane (Cytiva) by semi-dry Westen blotting. Next, the membrane was blocked for 1 h at RT in Tris buffered saline (TBS) + 0.1% Tween-20 (TBST, Tween-20 AppliChem) and 5% milk powder (Carl Roth). The primary antibodies were: p53 (Abcam, #131442), p21 (Cell signaling Technology, #2947), ß-Actin (Cell signaling Technology, #4970) diluted 1:2000 in blocking buffer and incubated overnight at 4 °C. After three washes with TBST, HRP-linked secondary antibodies (Cell Signaling Technology, #7074) were diluted 1:5000 in blocking buffer and incubated for one hour at RT, followed by washing and detection using enhanced chemiluminescence reagent (ECL, ThermoFischer Scientific) with a chemiluminescent imager (Azure 300, Azure Biosystems).

P53 and p21 expression were quantified relatively to ß-Actin expression normalized to the corresponding untreated control (Supplementary Fig. S7c; S8c, S11).

### Image-based dead/live assay

A normalized cell number was seeded, incubated for 4 days in full medium, then incubated for 2 h with 2 drops/ml culture NucBlue™ Live ReadyProbes™ Reagent (Hoechst) (ThermoFisher Scientific) and 4.5 μM propidium iodide (PI, Merck). For imaging a confocal Z stack was acquired at 2,5x magnification with a Cytation C10 microscope (Agilent). 24 h, 72 h and 120 h after 6 Gy irradiation, the staining and imaging was repeated. Nuclei and the area of the dead cell were determined from Z-projected images with the Gen5 software (Supplementary Fig. S9b, S9c).

### Flow cytometry-based cell cycle analysis

Organoids were dissociated into single cells and fixed with 100 μl 1% paraformaldehyde in PBS for 30 min at RT. 2 ml permeabilization buffer (0,1% Triton X-100 (Carl Roth, 3051.2) in 1x PBS) was added and incubated for 20 min. The cells were pelleted at 400 x g for 5 min at RT, washed twice with 2 ml 0,1% Tween 20 in 1x PBS (PBST) and blocked in PBST + 2% goat serum (Merck, G9023) for 30 min at RT, followed by incubation with anti-Ki67 eFluor™ 660 conjugated antibody (1:200; ThermoFischer Scientific, 50-5698-82) for 1.5 h at RT. Cells were washed and resuspended in FACS buffer with 10 μg/ml DAPI and incubated for 20 min at RT. DAPI signal and Ki67 expression were measured by flow cytometry (BD Bioscience, LSRFortessa™ flow cytometer). Data was analyzed and processed with FACSDiva™ Software FlowJo™ and R.

### Statistics

Statistics were calculated using Prism 10.1.2 (GraphPad) unless described separately. All error bars represent SD. Two-tailed unpaired t-tests were performed for the comparison of technical replicates. Comparisons between paired samples (genetically modified organoids and the corresponding control) were calculated using a paired t-test. *P*-values higher or equal 0.05 were considered as not significant (n.s.) (*: *p* < 0.05; **: *p* < 0.01; ***: *p* < 0.001; ****: *p* < 0.0001).

## Results

### Organoid biobank recapitulates the molecular and phenotypic characteristics of HNC

**Fig. 1:**
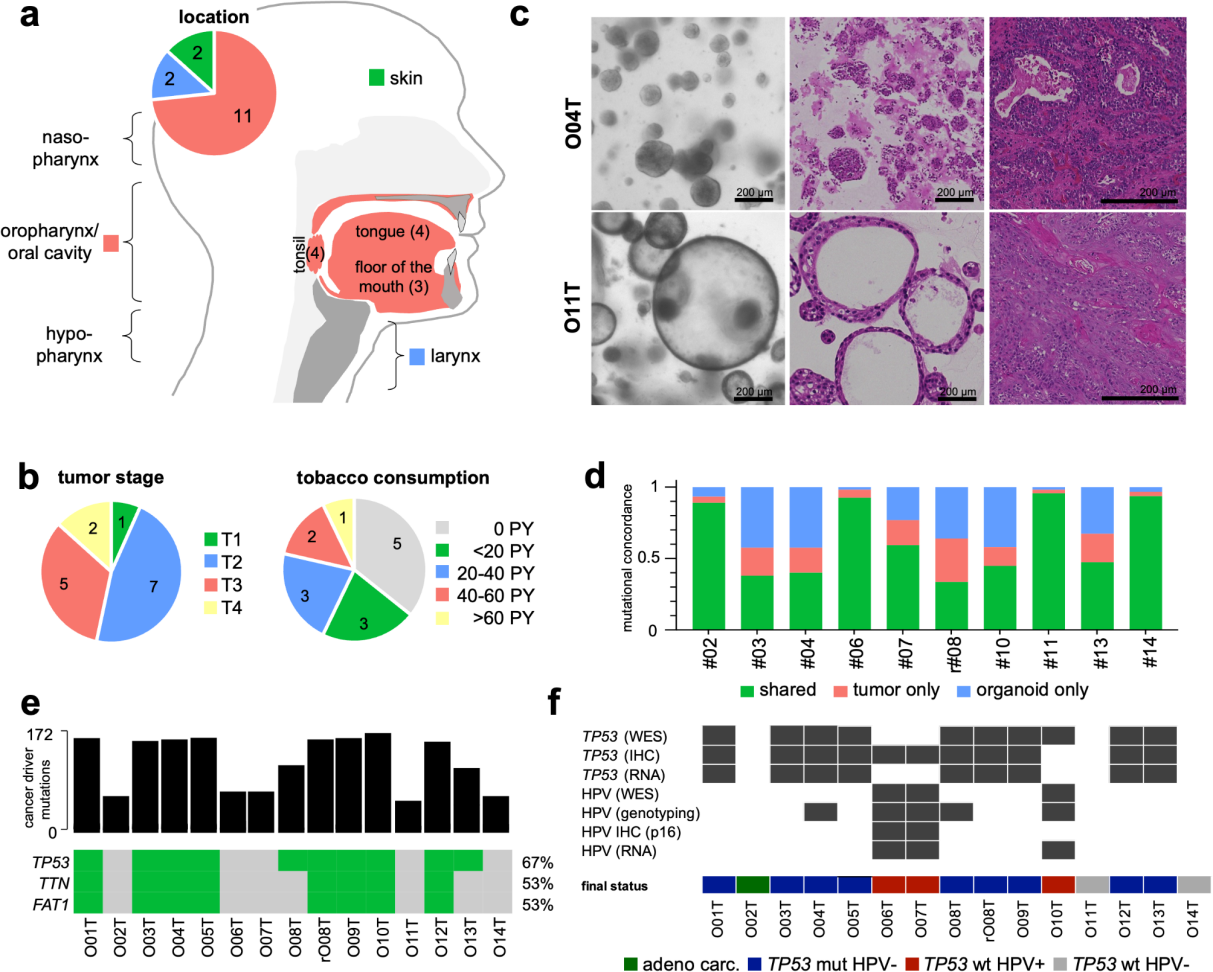
Clinical and genetic characteristics of head and neck cancer organoid biobank. (**a**) Overview of the tumor localizations and (**b**) clinical parameters of the experimental cohort. (**c**) Representative morphological images (left) and histology (H&E staining, middle) of two tumor organoid lines that grow compact (O04T) or cystic (O11T) and matched tumor histology (right). Scalebars are 200 μm. (**d**) Mutational concordance after whole exome sequencing (WES) of organoids and matched tumor tissues based on all detected SNPs > 0.05 VAF. (**e**) Detected number of cancer driver mutations and top recurrent mutations in PDTOs. (**f**) Evaluation of the *TP53*/HPV status in PDTO using WES, immunohistochemistry (IHC) and RNA sequencing. Additionally, the HPV status was determined by amplicon hybridization (VisionArray). The consensus classification for each PDTO is shown below

To generate the head and neck organoid biobank (HNOB), 15 tumor organoids from 14 patients (eight male and six female) of different stages, entities, and locations were established, reflecting the major HNSCC subtypes. One PDTO (O02T) was derived from an adenocarcinoma (Fig. 1a, b; Supplementary Fig. S1a; Supplementary Table S1). rO08T was an early recurrence of a tongue carcinoma three months after partial tongue resection without adjuvant treatment of the primary tumor O08T. All organoids were obtained by surgical resection without prior treatment, except for O12T collected after neoadjuvant chemoradiotherapy (CRTx) (Supplementary Table S1). In addition, three normal organoid lines were generated from unaffected tumor-adjacent regions of resection specimens (Supplementary Fig. 1b, Supplementary Table S1). Using adapted growth conditions from Driehuis et al. (2019) organoids could be long-term expanded (> 15 passages) [37]. Organoids showed exponential growth and were passaged on average every 7–14 days (Supplementary Fig. S1c). Furthermore, they maintained line-specific morphologies, mostly dense epithelial structures were observed apart from O11T and O11N, showing a more cystic phenotype (Fig. 1c; Supplementary Fig. S1a, b; S2).

For genomic characterization, ten PDTOs and corresponding tumor tissues were analyzed by whole exome sequencing (WES). Comparison of single nucleotide polymorphisms (SNPs) between PDTOs and tumor tissues revealed a good overall concordance of 63.4 ± 26.2% with comparable fractions of tumor- (13.3 ± 9.2%) and PDTO-specific SNPs (23.2 ± 18.1%) (Fig. 1d; Supplementary Table S2). Overall, most detected variants were missense mutations affecting C > T transitions (Supplementary Fig. S3a, b) as described previously for HNSCC [54]. Inspection of recurrent mutations confirmed the presence of known drivers (TCGA, Firehose Legacy, 2012) [49] (Fig. 1e). As expected, *TP53* was the most frequently mutated gene (in 10 of 15 PDTOs). Two common loss-of-function variants were detected: *TP53*^*R248Q*^ (in O01T, O03T, O04T; O05T, rO08T, O09T, O10T, O12T and O13T) and *TP53*^A138V^ (in O08T and O13T) (Supplementary Fig. S3c). R248Q affects the DNA binding region, disabling target gene activation by p53 tetramers [55]. *TP53*^A138V^ is a more rare and clinically less reported variant [56]. *TTN* and *FAT1* mutations (8 out of 15) could always be detected in combination with *TP53* mutations. All three mutations have been associated to a high-risk subgroup of HNSCC [57–60]. Depending on the *TP53* status, significant differences in the detected tumor mutations were observed (*p* = 0.033), compatible with the well-described role of p53 in preserving genome integrity (Fig. 1e; Supplementary Fig. S3d). Consistently, a side-by-side comparison of the two organoid lines O08T with the early recurrence rO08T showed increased number of detected alterations. Concordance analysis revealed 33.2% shared mutations between O08T and rO08T. O08T had 29.5% and rO08T had 37.3% private mutations, indicating a genetic shift during therapy.

### Functional classification of the organoid biobank regarding the TP53 and HPV status

To address consistency of the pathological subtype classification, PDTOs were analyzed according to *TP53* and HPV status. p53 IHC staining revealed positivity in 11 PDTOs and was confirmed by WES in 10 PDTOs (Supplementary Fig. S4a). *TP53* mutant PDTOs showed significantly higher level of p53 staining compared to *TP53* WT PDTOs (Supplementary Fig. S4b). *TP53* mutations detected by WES were confirmed on the transcriptomic level in 8 out of 9 PDTO (Fig. 1e, f; Supplementary Fig. S3e). In O10T, a *TP53* mutation was detected in WES in passage 3, that could not be confirmed in p53 IHC and RNAseq in passage 6 indicating a genotypic drift during prolonged culture followed by a phenotypic change (Fig. 1e, f; Supplementary Fig. S1).

The HPV status of all PDTOs was determined by IHC staining of the surrogate marker p16, genotyping by amplicon hybridization (VisionArray), WES and RNA sequencing (Fig. 1f; Supplementary Fig. S5; Supplementary Table S3). While in tissues, HPV 16, HPV 33 and HPV 35 could be detected by amplicon hybridization, only HPV 16 was found in PDTOs (Supplementary Table S3) and all organoids from normal adjacent tissue were HPV – (Supplementary Table S3). Of note, amplicon hybridization showed positivity for lines that could not be confirmed by IHC or sequencing, indicating false positive results (Fig. 1f).

Together, the analysis led to the classification of 9 *TP53* mutant and three HPV+ PDTOs that occurred in a mutually exclusive manner as previously described [61]. In addition, two PDTOs (O11T and O14T) were classified as both HPV – and *TP53* wildtype (Fig. 1f). These results indicate that the accuracy of pathological assessment can be increased by combined histological and molecular analysis in tumor tissues and PDTOs.

### TP53 deficient and HPV+ PDTOs show characteristic transcriptomic features

**Fig. 2:**
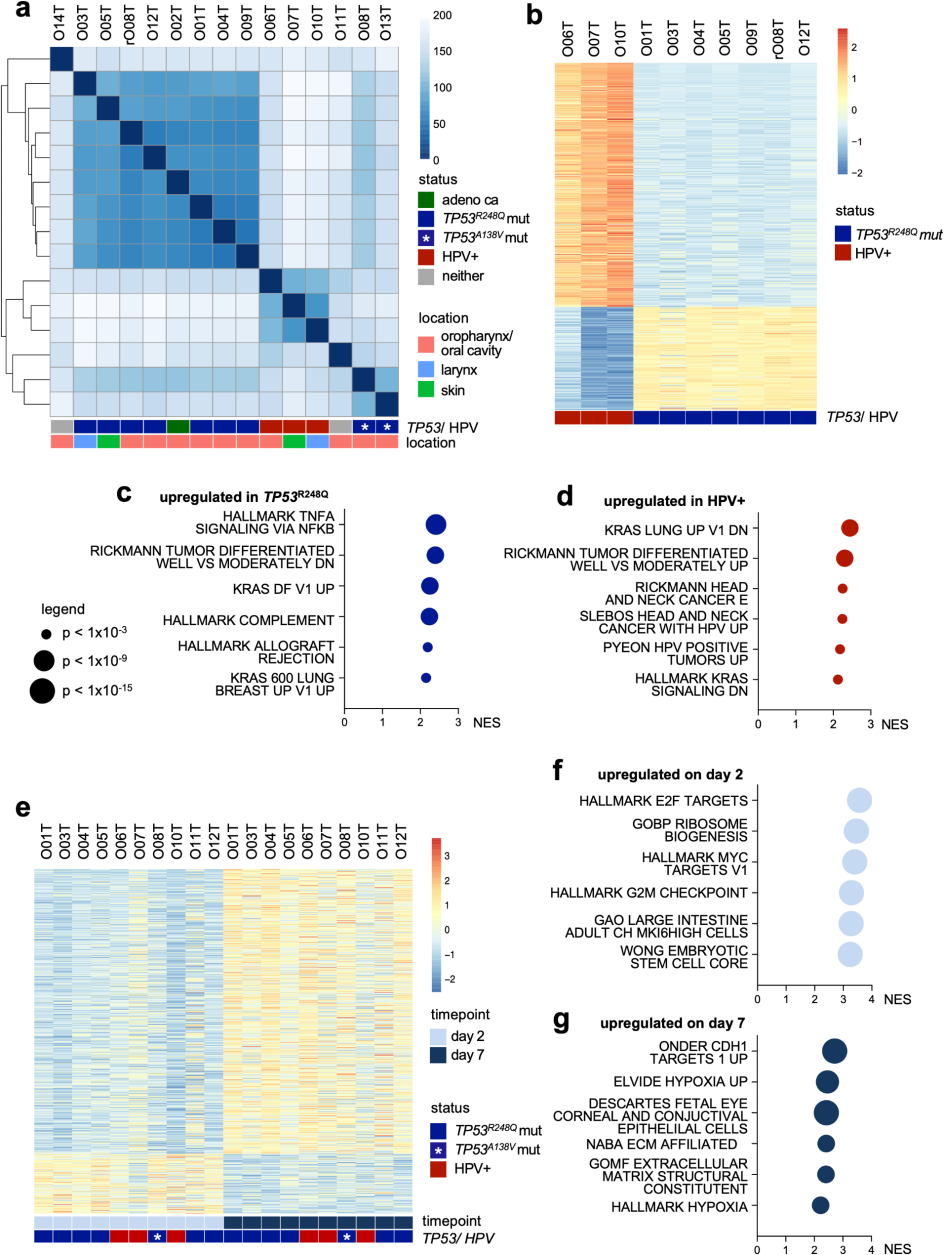
Transcriptomic characteristics of head and neck cancer organoid biobank. (**a**) Heatmap of transcriptome-wide similarity scores of all 15 PDTO models seven days after seeding. *TP53*/HPV status and tumor localization are shown. Unsupervised hierarchical clustering shows distinct subtypes (note that *TP53*^*R248Q*^ and *TP53*^*A138V*^ mutations are found in different clusters). (**b**) Heatmap of differentially expressed genes according to *TP53*/ HPV status (only *TP53*^*R248Q*^ mutant organoids were included). (**c**) Upregulated pathways in *TP53*^*R248Q*^ mutant and (**d**) HPV+ organoids. GSEA of MSigDB signatures with normalized enrichment scores (NES) is shown. Size indicates p-value from adjusted t-tests. Raw data are shown in Supplementary Tables S4a and S4b. (**e**) Heatmap of differently expressed genes after distinct times of culture (day 2 vs. day 7 after seeding). *TP53*/HPV status and timepoint are shown. (**f**) Upregulated pathways in organoids on day 2 and (**g**) day 7. Data shown as in (**c**/**d**). Raw data are shown in Supplementary Tables S5a and S5b

For transcriptomic profiling of PDTOs bulk RNA sequencing was performed. Correlation analysis, unsupervised hierarchical clustering and principal component analysis resulted in distinct clusters of *TP53* mutant (separating *TP53*^*R248Q*^ and *TP53*^*A138V*^ mutations) and HPV+ organoids (Fig. 2a; Supplementary Fig. 3e). The adenocarcinoma line (O02T) showed similarity to the *TP53*^*R248Q*^ mutants. No clustering according to anatomical location was observed. Next, differential gene expression analysis was performed between HPV+ and *TP53*^*R248Q*^ mutant PDTOs (Fig. 2b; Supplementary Table S4a). Gene set enrichment analysis (GSEA) showed that *TP53* mutation is linked with significant upregulation of proinflammatory Hallmark signatures (TNF/NF-κB signaling, complement activation, allograft rejection) and epithelial-mesenchymal transition (EMT) that is associated with a more malignant phenotype in HNSCC [62, 63] (Fig. 2c; Supplementary Table S4b). Moreover, HPV+ PDTOs showed known HPV signatures, downregulation of KRAS signaling (Fig. 2d) and upregulation of glutathione transferase that was reported previously as E7 induced pro-survival program [64].

To assess the impact of cell differentiation on gene expression, RNA sequencing was performed at day 2 and day 7 day after splitting, followed by differential gene expression analysis (Fig. 2e; Supplementary Table S5a, b). On day 2, GSEA showed enrichment of signatures related to proliferation (E2F targets, MYC targets, G2M checkpoint, stemness and ribosome biogenesis; Fig. 2f). In contrast, differentiation-associated signatures including hypoxia and extracellular matrix were upregulated at the late timepoint (Fig. 2g) irrespective of the *TP53*/HPV status, indicating the prominent capacity of HNSCC organoids to undergo spontaneous differentiation.

### The HNOB shows heterogenous growth and sensitivity to *TP53* stabilization

**Fig. 3:**
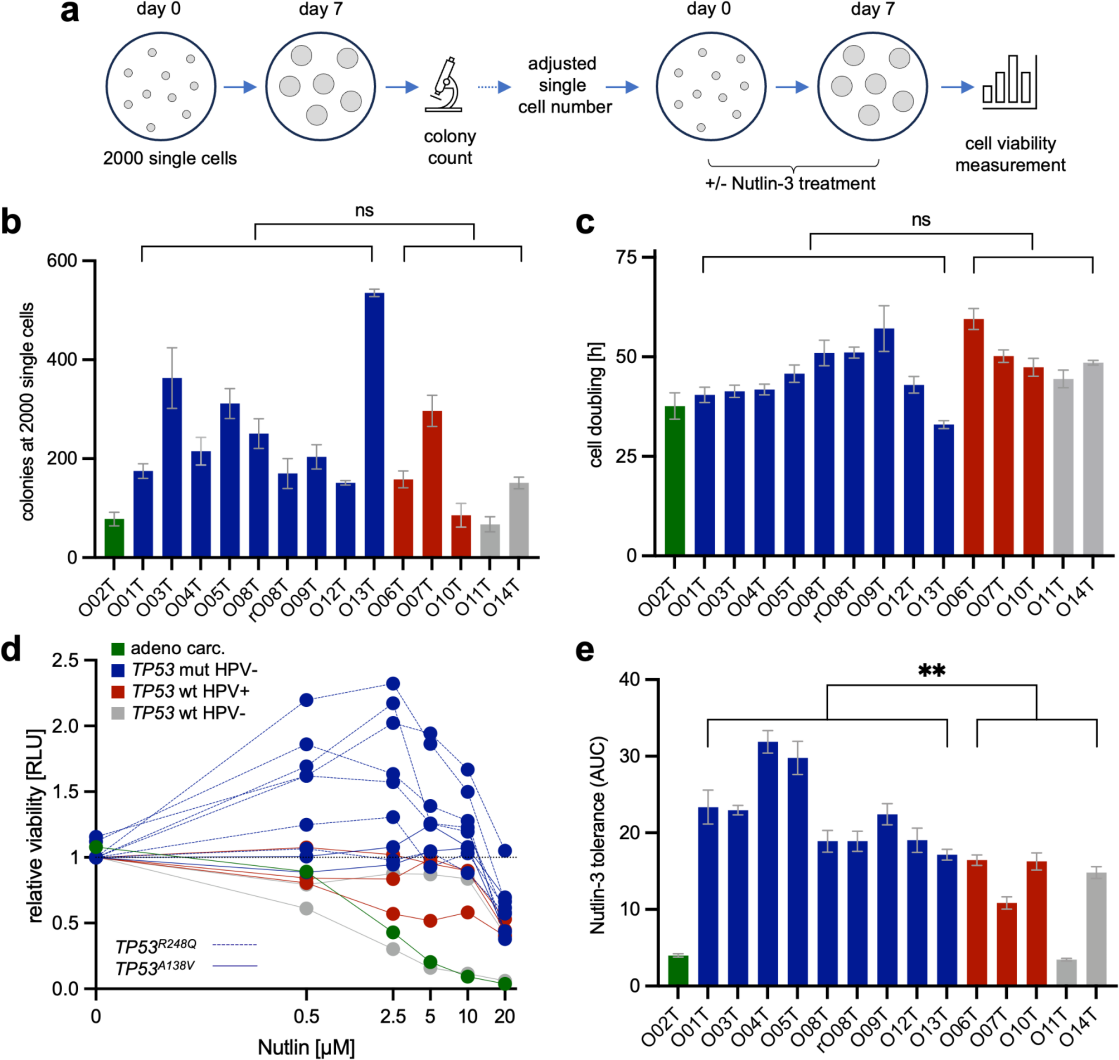
Phenotypic characterization of the head and neck cancer organoid biobank. (**a**) Experimental setup of colony formation assay. Colony number was counted 7 days after single cell seeding. Subsequently, normalized cell numbers were seeded for the Nutlin-3 tolerance assay before cell viability measurements using CellTiterGlo™ (in *n* = 3 wells each). (**b**) Number of colonies 7 days after seeding 2000 single cells. Non-significant (ns) differences between *TP53* mut and wt lines (t-test). (**c**) PDTO cell doubling rate. RealTimeGlo™ measurement was conducted over 144 h (in *n* = 6 wells each). (**d**) Nutlin-3 dose response. Viability assay using CellTiterGlo® 7 days after seeding of adjusted single cell numbers was measured in triplicates. Note that low and intermediate Nutlin-3 concentrations increase ATP levels in the *TP53*^*R248Q*^ PDTOs (dashed lines). (**e**) Area under the curve (AUC) of Nutlin-3 tolerance using CellTiterGlo®. Statistical analysis (t-test) shows significantly higher ATP levels in *TP53* mutant PDTOs compared to *TP53* wt (*p* = 0.004)

For functional characterization, the organoid formation and growth capacity were determined. After seeding 2000 single cells and culture for 7 days, prominent differences in colony formation were observed that were not significantly associated with the *TP53*/HPV status (Fig. 3a, b). For all subsequent experiments, the number of single cells was normalized to obtain comparable organoid numbers. RealTimeGlo™ measurement was conducted over 144 h to quantify cell expansion, which showed comparable cell doubling times among the lines (Fig. 3c). Thus, colony formation and growth are subtype-independent characteristics. Subsequently, TP53 activity was assessed by treatment with MDM2 inhibitor Nutlin-3. As expected, *TP53* mutant PDTOs showed reduced Nutlin-3 sensitivity (Fig. 3d, e; *p* = 0.004), confirming our subtype classification. Interestingly, low and intermediate Nutlin-3 concentrations even caused increased ATP levels in the *TP53*^*R248Q*^ PDTOs (Fig. 3d). In contrast, *TP53*^*A138V*^ PDTOs showed resistance to exposure to Nutlin-3, but no increased ATP levels, indicating functional heterogeneity between the *TP53* alleles, with R248Q exerting an oncogenic effect when stabilized moderately.

### In vitro assessment of individual radiation responses

**Fig. 4:**
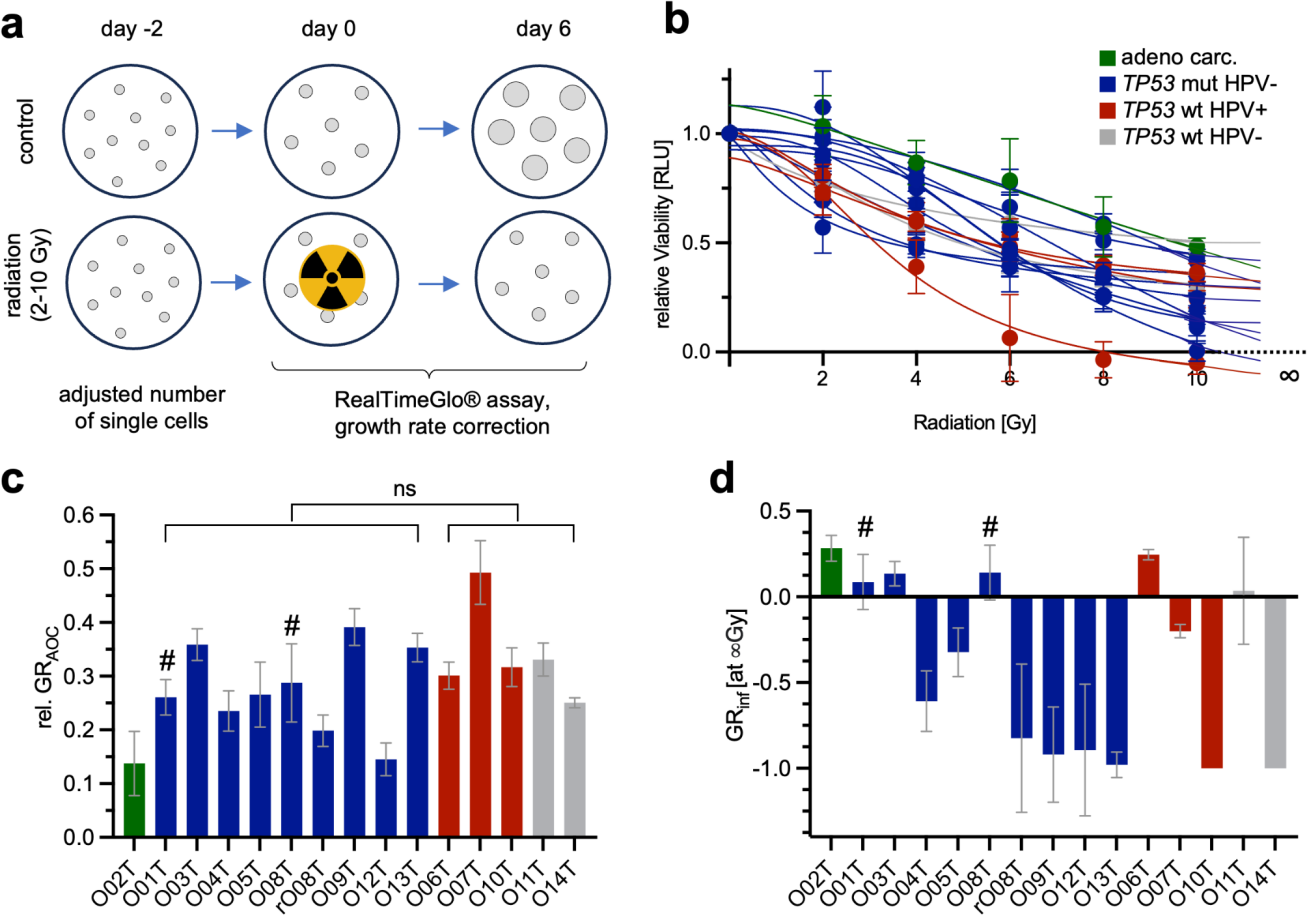
Radioresponse (RR) of head and neck cancer organoid biobank. (**a**) Scheme of radioresponse (RR) assay. Adjusted single cell number was seeded and irradiated after 48 h. The RR to 0, 2, 4, 6, 8 and 10 Gy was measured 144 h after radiation using RealTimeGlo™ (in *n* = 6 wells each). (**b**) Evaluation of the RR. Growth rate (GR) corrected values 144 h after single irradiation. **c**/**d**) Summary of RR data. Patients that showed a subsequent clinical relapse were marked (#). No significant differences were observed with regard to *TP53*/ HPV status. (**c**) area over the curve and GR_inf_ (**d**) at an imputed infinite dose. Negative GR_inf_ values indicate cytotoxic response, 0 indicates a cytostatic response and positive values indicate persistent growth. Error bars show minimum and maximum values

In clinical practice, HNC patients show a highly heterogeneous response to radiotherapy. Consequently, there is an urgent need for preclinical assays to study these differences and perform individualized therapy testing. In the HNOB, the radio response (RR) was evaluated after single dose irradiation (2 to 10 Gy; Fig. 4a). Cell viability was monitored using RealTimeGlo® assay and calculated using growth rate (GR) metrics [65] to reflect growth differences among the lines, which closely mirrored endpoint measurements (Supplementary Fig. S6a, b). Two characteristic phenotypes were observed: lines that showed complete response at higher doses and lines that partially responded to low doses but persisted at higher doses (Fig. 4b). Quantification of the area over the curve (GR_AOC_) showed prominent differences among the lines (Fig. 4c). In particular, the adenocarcinoma PDTO showed a high radiation tolerance, consistent with clinical observations [66]. There was no significant association between tumor entity or anatomic location and GR_AOC_. However, in clinical routine, an easy and clear classification into good and poor responders would be desirable. For this purpose, we determined the GR_inf_ at a simulated infinite dose (Fig. 4d). Six of the 15 PDTOs showed positive GR_inf_ values, indicating persistent growth at high dosage, which we used as a classifier for high-risk cases. Of the 14 different patients in the HNOB, 10 received adjuvant RT/CRTx after primary surgical resection (Supplementary Table S1). Two of them had a recurrence within the study period (P01, P08) that both showed positive GR_inf_ values and were therefore correctly classified as high-risk patients. All other tumors classified as poor responders in the organoid model could be resected *in sano* with large safety margins. In none of the lines classified as good responders, a clinical relapse occurred during the follow-up period of at least two years. In summary, our experiments showed a strong heterogeneity of the RR that was linked with individualized clinical outcomes indicating utility for personalized testing.

### Modeling *TP53* mutation and HPV infection in normal and tumor organoids

**Fig. 5:**
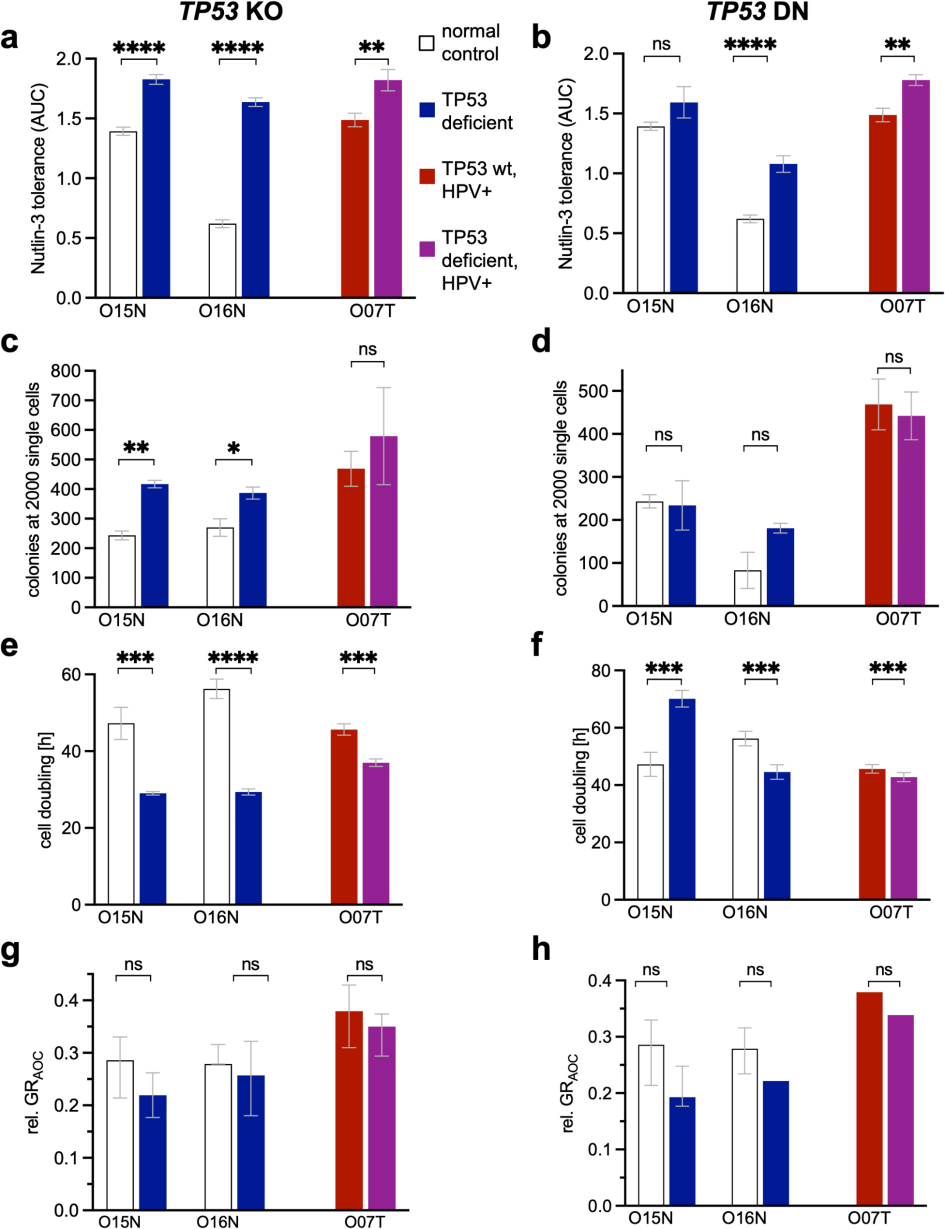
Genetic *TP53* perturbations promote Nutlin-3 resistance and cell growth. Functional characterization of CRISPR/Cas9-induced *TP53* knock-out (ko; **a**,**c**, **e**,**g**) and lentiviral expression of dominant negative p53 (DN; **b**,**d**, **f**,**h**) in normal and tumor organoids. For statistical caparisons (t-tests) the individual control organoids were used. **a**/**b**) Nutlin-3 tolerance assay. Dose responses (AUC) were measured in triplicates using CellTiter-Glo™. **c**/**d**) Colony formation assay. Organoid number 7 days after seeding 2000 cells (in *n* = 3 wells each). **e**/**f**) Cell doubling time measured with RealTime-Glo™ (in *n* = 5 wells each) over the course of 6 days. **g**/**h**) Radiation response. The relative cell viability 144 h after radiation (0 to 10 Gy) compared with initial viability was measured in *n* = 6 wells each using RealTimeGlo™. Growth rate corrected area over the curve data (GR_AOC_). Error bars show minimum and maximum values

To study tumor progression and evaluate the direct impact of the *TP53* and HPV status, we established protocols for genetic modification. First, the effect of *TP53* loss was studied in normal organoids from tumor adjacent tissue (O15N, O16N) and a tumor organoid (O07T) that was *TP53* wildtype and HPV+. *TP53* was either ablated by CRISPR/Cas9 mediated gene deletion (Supplementary Fig. S7a) or by lentiviral introduction of the *TP53 DN* variant R248Q (Supplementary Fig. S8a). Loss of *TP53* function was selected by addition of Nutlin-3 and Cas9-induced indels were confirmed by Sanger sequencing (Supplementary Fig. S7b). Dose titration experiments showed Nutlin-3 resistance (Fig. 5a, b). Western blot analysis showed that p53 expression was increased upon overexpression but only weakly reduced in Cas9-modified lines, indicating induction of heterogenous mutations that may not influence the protein level (Supplementary Fig. S7c, S8c). However, a reduced induction of the p53 target p21 after Nutlin-3 treatment showed the functional deficiency of the pathway. *TP53*-deficient normal organoids formed more colonies from single cells than corresponding control lines (Fig. 5c, d). In most tumor and normal organoids, the cell doubling time was significantly lower than their control lines (Fig. 5e, f). The GR_AOC_ showed a slight decrease in both normal and tumor organoids, indicating that *TP53* loss alone is not sufficient to confer radiation resistance (Fig. 5g, h).

**Fig. 6:**
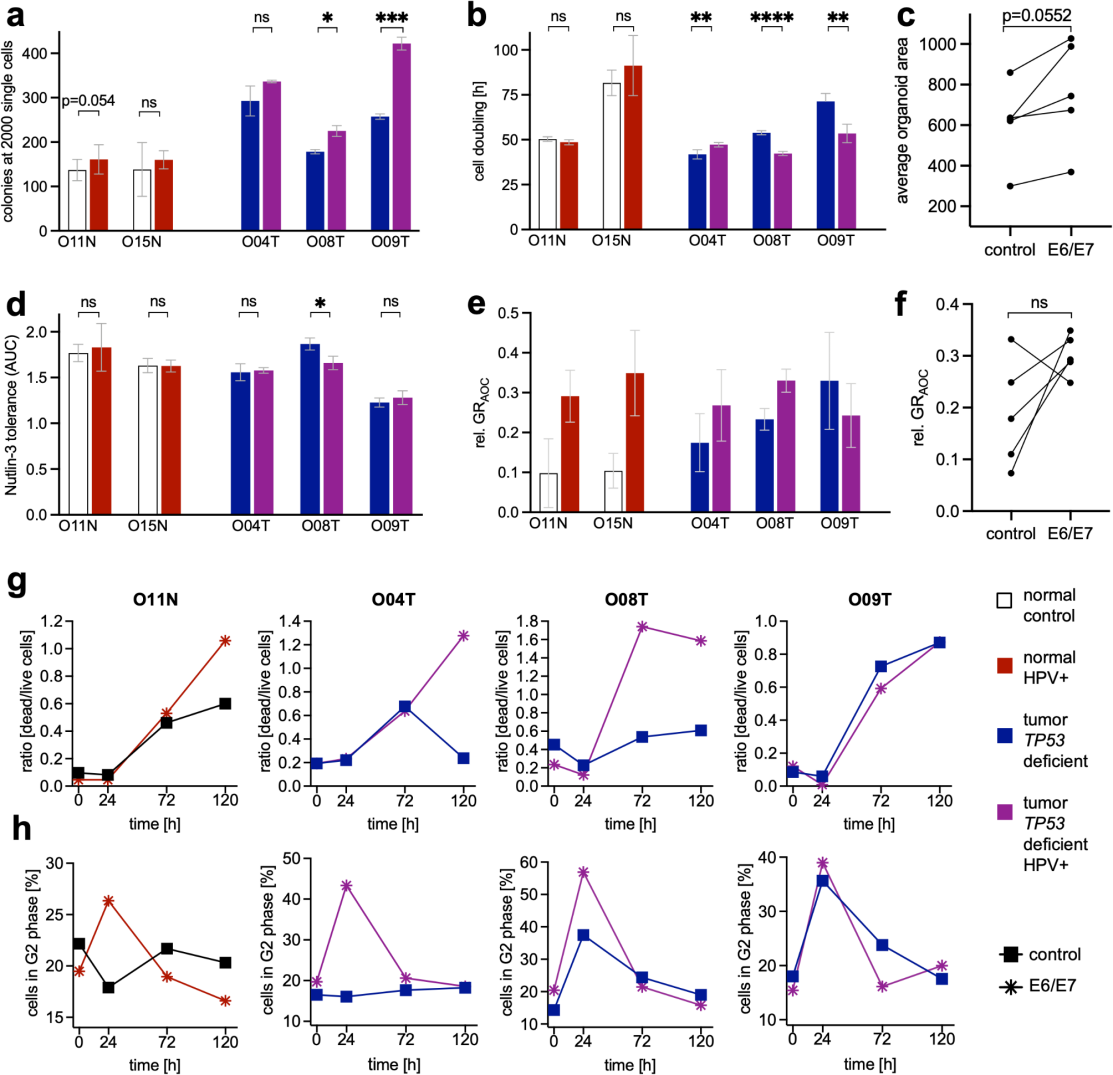
Modelling *HPV* by lentiviral E6/E7 expression induces cell growth and radiosensitivity. Characterization after lentiviral E6/E7 overexpression in normal and tumor head and neck organoids. All organoids were HPV – before transduction. For statistical caparisons (t-tests) matched controls were analyzed. (**a**) Colony formation assay. Organoid number 7 days after seeding 2000 cells (in *n* = 3 wells each). (**b**) Cell doubling time measured with RealTime-Glo™ (in *n* = 5 wells each) over the course of six days. (**c**) Average area of organoids measured on morphologic images (mean values from *n* = 12 wells each). (**d**) Nutlin-3 tolerance assay. Dose responses (AUC) were measured in triplicates using CellTiter-Glo™. (**e**) Radiation response. The relative cell viability 144 h after radiation (0 to 10 Gy) compared with initial viability before radiation was measured in *n* = 6 wells each using RealTimeGlo™. GR_AOC_ and minimum and maximum values are shown. (**f**) Statistical comparison of GR_AOC_ in all lines (paired t-test). (**g**) Live/dead cell assay. Adjusted single cell number was seeded and irradiated with 6 Gy on day 4. Organoids were incubated with Hoechst and PI at 0 h, 24 h, 72 h, and 120 h after irradiation. Image-based measurement of the dead/live ratio in control and E6/E7 expressing lines. For details see Fig. S9b and c. (**h**) Flow cytometry-based cell cycle assay. Performed as in g) followed by organoid dissociation and staining with DAPI and anti-Ki67. For more details, see Fig. S10a-d

To investigate the phenotypic consequences of HPV infection, the oncogenes E6 and E7 from the high-risk strain HPV 16 were introduced using a lentiviral strategy [53] (Supplementary Fig. S9a) in two normal tissue organoid lines (O11N, O15N) that were negative in HPV genotyping (Supplementary Table S3). Additionally, *TP53* mutated (HPV–) tumor organoids (O04T, O08T, O09T) were used to study the effect of the combined presence of both drivers that have been described to rarely coexist [61]. E6/E7 overexpression was confirmed by RNA sequencing (Supplementary Table S3). In two of three tumor organoids, but not in normal organoids E6/E7 caused significantly increased colony formation (Fig. 6a) and reduced the cell doubling time (Fig. 6b). The organoid size was generally increased upon E6/E7 expression (*p* = 0.052) (Fig. 6c). Nutlin-3 sensitivity was not consistently affected (Fig. 6d), indicating that E6 mediated TP53 destabilization [67] does not play a major role in primary cells from the oral epithelium. However, similar as observed by engineering of *TP53* status, E6/E7 overexpression confirms the possibility of co-existence of both drivers.

Next, the influence of HPV on the RR was investigated. Dose titration showed a strongly increased sensitivity (GR_AOC_) in both normal and in 2 of 3 tumor lines (Fig. 6e and f). To study the underlying mechanism, the life/dead cell ratio was measured using PI/Hoechst staining and confocal imaging (Supplementary Fig. S9b, S9c). After a single dose irradiation with 6 Gy, we observed that E6/E7 expression was associated with a progressively increased fraction of dead cells in normal (O11N) and tumor organoids (O08T), or a failure to recover from radiation exposure (O04T) (Fig. 6g). Only in O09T, in which E6/E7 expression did not confer radiosensitivity, no discernable effect on cell death was observed. Next, the cell cycle distribution was studied by flow cytometry after DAPI and KI67 immunostaining (Supplementary Fig. S9a-d). In the absence of radiation, no changes of the cell cycle were observed in E6/E7 expressing lines. However, 24 h after 6 Gy irradiation, the radio-sensitive lines showed a strikingly increased G2 population (Fig. 6h). 72 and 120 h after irradiation, all lines had returned to the baseline cell cycle distribution, indicating a transient, E6/E7 mediated G2 arrest.

## Discussion

For risk stratification and to enable individualized treatments, molecular diagnostics has become essential in modern oncology. Targeted therapies are available for many tumor entities, which has led to increased survival and reduced side effects [68–72]. In HNSCC, several potential biomarkers have been identified [10, 73–75]. However, in clinical practice, to date only HPV diagnostics has been implemented [76]. Although this allowed to test de-escalation strategies in clinical studies [23, 26], the underlying molecular mechanisms are not sufficiently understood and in particular for HPV – tumors therapy prediction remains a challenge.

Here, we report the establishment of the *head and neck organoid biobank* (HNOB) to evaluate HNC PDTO models as a predictive tool and to gain mechanistic insights into *TP53*/HPV-driven tumors. By comparing orthogonal TP53/HPV tests used in clinical routine including IHC- and DNA/RNA-based methods, we observed discrepancies for HPV detection, which can result from use of surrogate markers or amplification-based methods. We found that a combined molecular characterization improves the accuracy of the pathological assessment. Bulk RNA sequencing analysis proofed most robust for detection of HPV, avoiding false positive results. This is consistent with recent meta-analyses that have stressed the importance of RNA-based diagnostics to distinguish bystander infection from HPV-driven cancers [21, 22]. Furthermore, we observed distinct transcriptomic subtypes depending on the HPV-status and the presence of specific *TP53* mutant alleles, including the dominant-negative *TP53*^*R248Q*^ mutation that is known for high motility and invasiveness in oral squamous cell carcinoma [77]. These results confirmed that PDTOs maintain key clinical features ex vivo, allowing the derivation of oncogene-specific expression signatures. Also, we observed prominent temporal changes of gene expression that occur during culture of HNSCC organoids, reflecting the high potential for spontaneous differentiation. Together, these organoid-derived signatures will help to interpret clinical gene expression data.

PDTOs could play a key role for clinical decision-making and help to predict if patients benefit from radiotherapy, a treatment modality that can also cause severe morbidity and functional impairment. For functional characterization, we developed informative assays that capture the phenotypic heterogeneity among PDTO. Whereas proliferation and colony formation were not associated with the presence of specific oncogenic drivers, Nutlin-3 sensitivity served as a reliable indicator of *TP53* deficiency. In addition, we developed a 3D assay for live cell monitoring of the RR and found that the GR_inf_ can serve as a read-out to discriminate PDTO models that show resistance to high doses of radiation. Clinical follow-up showed that this indicator may help to identify patients at high risk of relapse. In contrast, none of the 9 patients with organoids that displayed GR_inf_ values < 0, showed any relapse in an over 2 years follow-up period. In contrast, individual risk stratification was not possible by only using *TP53*/HPV status. Most likely, the overall RR is influenced by additional somatic alterations, chromosomal instability and pathway activities including the NF-κB signalling [1, 78, 79]. While our results are promising, the current cohort is too small to cover rare HNC subtypes. In future, the investigation of larger cohorts and longer follow-up periods is required to validate our findings. Co-clinical studies should validate the predictive power of our risk stratification model, most optimally as part of prospective, multicenter trials.

The molecular and phenotypic heterogeneity of HNC is well-reflected by the HNOB. However, to gain mechanistic insights into role of specific drivers, we analyzed genetically engineered organoids: *TP53* loss as well as HPV increased colony formation and cell doubling speed in normal and tumor organoids. *TP53*-deficient HNC is clinically associated with a poor RR and high risk of relapse [1, 55, 80]. Interestingly, we did not observe increased RR in our organoid lines after CRISPR/Cas9 disruption of *TP53* or dominant negative overexpression, which argues against a direct causal relationship. Instead, the poor patient outcome may be caused indirectly by induction of secondary mutations. This was supported by the increased tumor mutation number detected in *TP53* deficient PDTOs (Fig. S3d). Consistently, HNSCC patients with *TP53* mutant tumors present at a higher age and often after long periods of mutagenic exposure to nicotine and/or alcohol compared to HPV+ patients [1]. In contrast, HPV E6/E7 oncogene expression induced radiation sensitivity that was associated with a pronounced, but transient G2 arrest. Our results are consistent with published data from primary oral epithelial cells in 2D culture [81]. We conclude that oncogene-mediated cycle promotion induces a new vulnerability, which may explain the favorable clinical response of HPV positive tumors [1, 23] and could create therapeutic opportunities. Interestingly, similar observations were made in models that combined both oncogenic drivers. This is in contrast to the rare incidence of co-occurrence in clinical samples [5, 82] and argues against the hypothesis of a functional incompatibility between the HPV and *TP53* mutations. Together, our study shows how a characterized organoid biobank can provide mechanistic insights on *TP53*/HPV-driven HNSCC, which could enable more precise clinical decision-making in the future.

## Conclusions

Our findings demonstrate the translational value of head and neck organoid models as a strong resource of cancer research. RR testing using PDTO models has the potential to play a key role for clinical decision-making. PDTOs should be implemented in large clinical trials for validation. Genetically modified HNC organoids enable to study the direct impact of *TP53*-loss and HPV infection, the main drivers in HNC. These results improve mechanistic understanding of HNC and provide functional and molecular biomarkers for HNC subtypes.

## Electronic supplementary material

Below is the link to the electronic supplementary material.

Supplementary Material 1

Supplementary Material 2

## Data Availability

Patient-derived organoids and materials can be provided upon reasonable request and following approval by the institutional review/ ethics board of the University Cancer Center Frankfurt (UCT). The RNA and Exome sequencing data will be deposited to the European Genome-Phenome Archive (www.ebi.ac.uk/ega/).

## Abbreviations

AOC: Area Over the Curve
AUC: Area Under the Curve
CA: Carcinoma
CM: Conditioned media
CRTx: Chemoradiotherapy
CTG: CellTiter-Glo™
DMSO: Dimethylsulfoxide
DN: Dominant negative
FACS: Fluorescence-activated cell sorting
FFPE: Formalin-fixed paraffin-embedded
GR: Growth rate
Gy: Gray
H&E: Hematoxylin and Eosin
HNC: Head and neck cancer
HNOB: Head and neck organoid biobank
HNSCC: Head and neck squamous cell carcinoma
HPV: Human papillomavirus
IHC: Immunohistochemistry
ko: Knockout
RNAseq: RNA sequencing
RR: Radio response
RT: Radiotherapy
RTG: RealTime-Glo™
SNV: Single nucleotide variants
TP53: Tumor Protein 53
VAF: Variant allele frequency
WB: Western Blot
WES: Whole exome sequencing
WT: Wildtype
