# Supplementary material for: Head and neck tumor organoid biobank for modelling individual responses to radiation therapy according to the TP53/HPV status
Source: J Exp Clin Cancer Res. 2025 Mar 5;44:85. doi: 10.1186/s13046-025-03345-3 (PMC11881459; doi:10.1186/s13046-025-03345-3)
Supplement: Supplementary file 1 — Supplementary Material 1 [file 13046_2025_3345_MOESM1_ESM.docx]

**Supplemental information titles and legends**

**
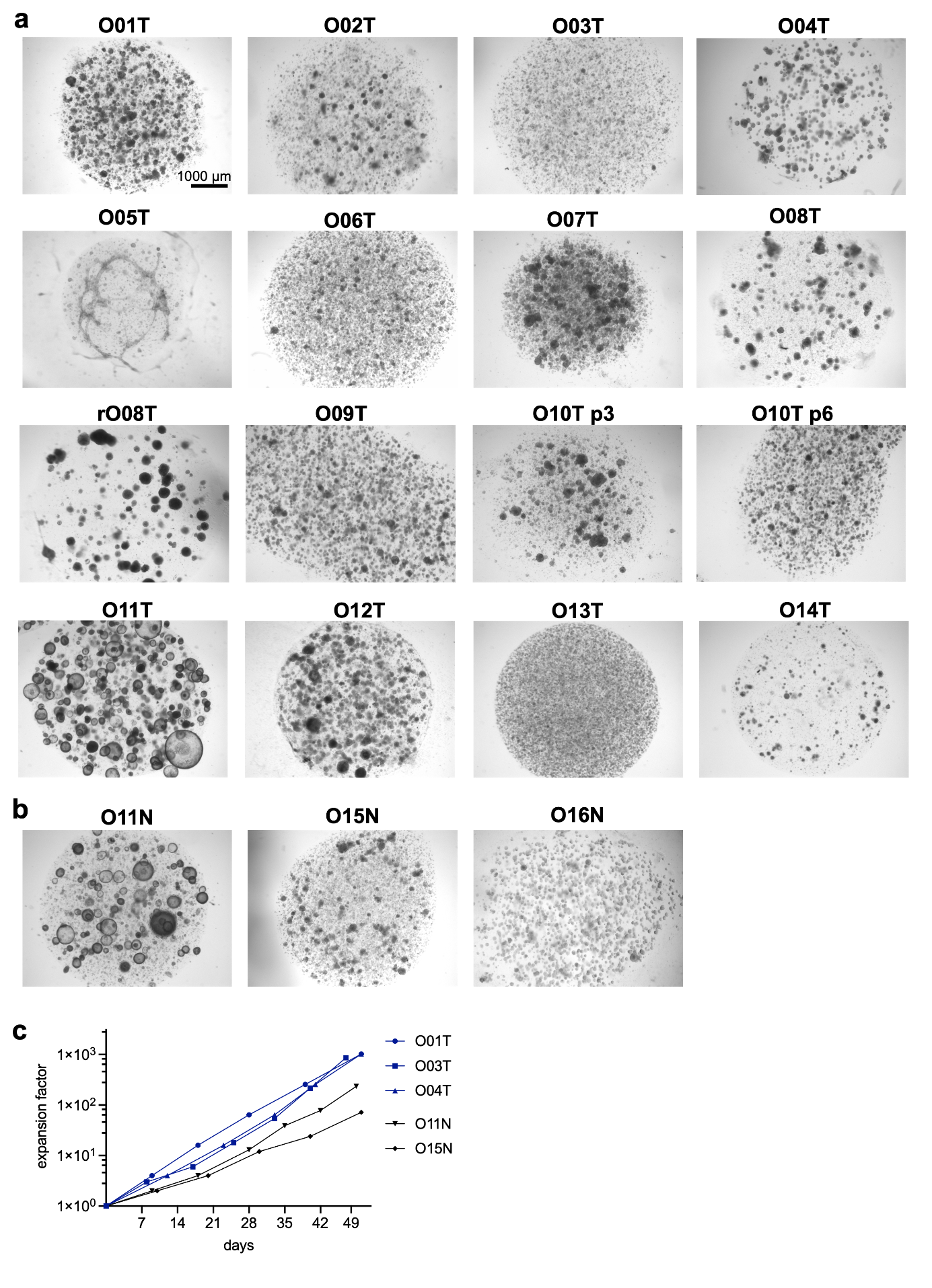
**

**Fig. S1｜HNSCC organoid morphology and expansion.**

a) Morphological images of tumor organoids and b) normal organoids derived from patients. All images were captured using a 2× objective 7 days after seeding single cells. Scalebar is 1 mm. O10T has changed the phenotype over prolonged cultivation. Morphological images from passage 3 (p3) and passage 6 (p6) are shown. c) Growth curve of representative organoid lines over the course of 7 weeks.

**
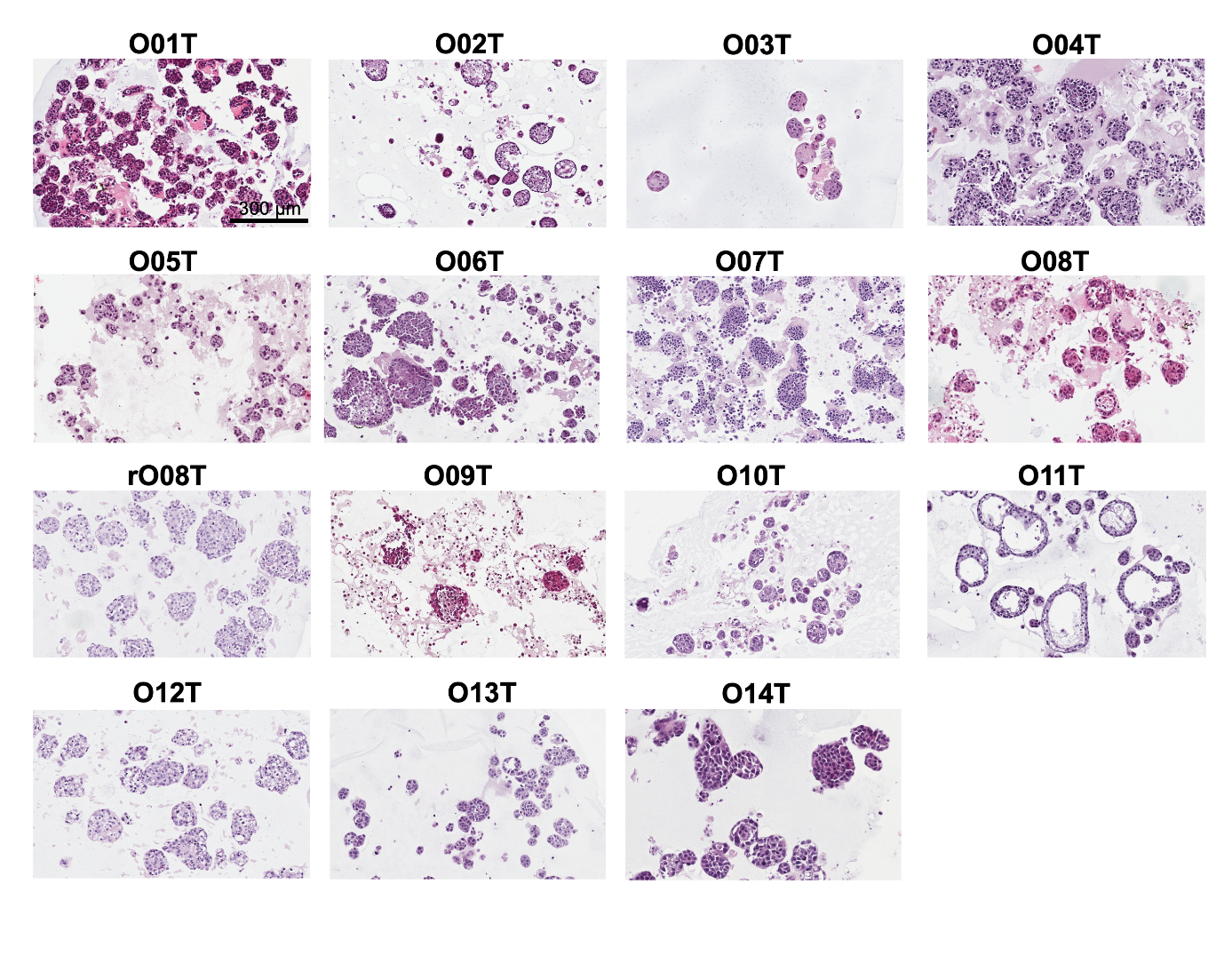
**

**Fig. S2｜Hematoxylin and eosin staining of HNSCC organoids.**

Representative hematoxylin and eosin staining of the PDTO models 7 days after seeding single cells. Images were captured with a 20× objective. Scalebar is 300 µm.

**
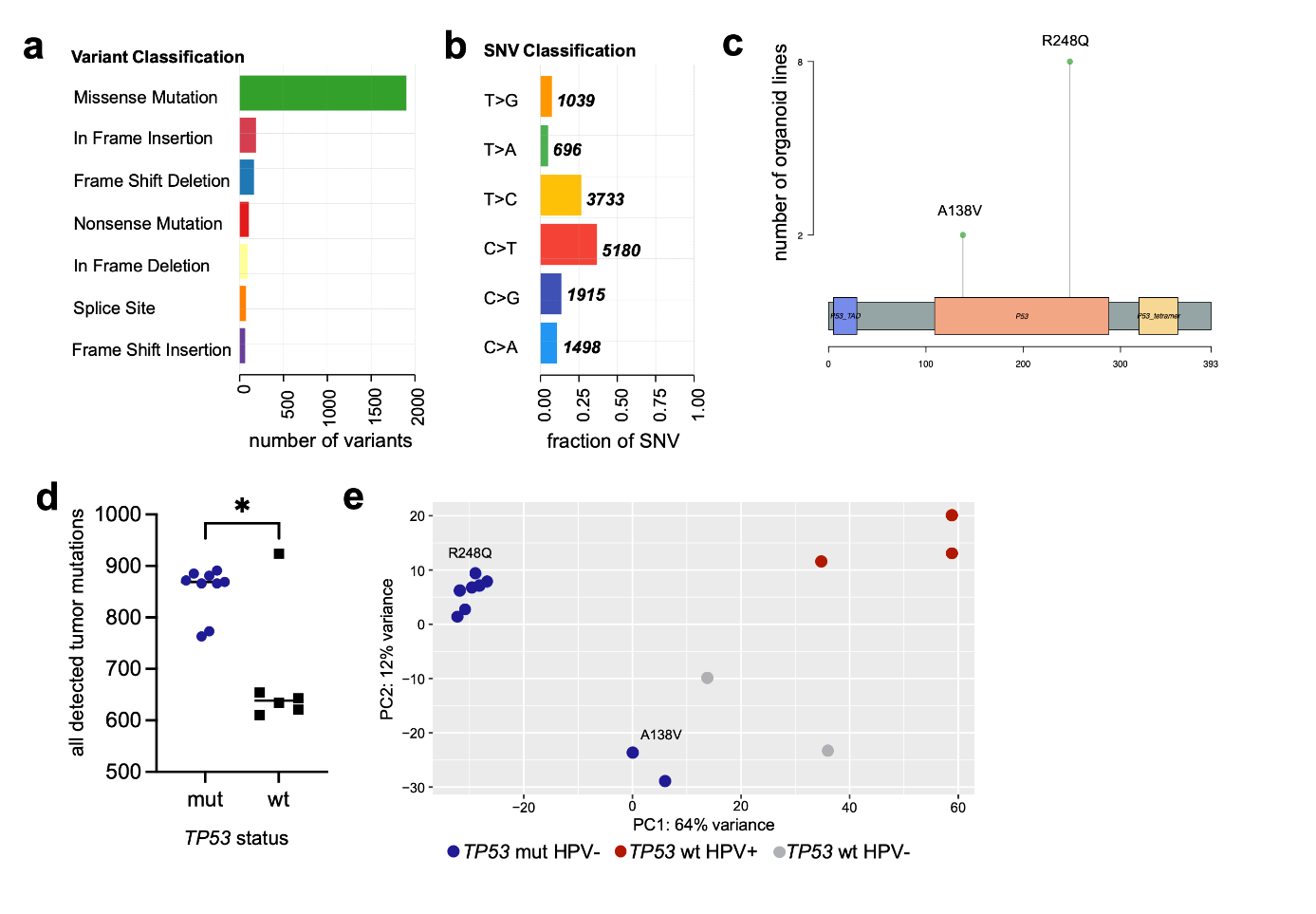
**

**Fig. S3｜Characterization by whole exome sequencing (WES) and RNA sequencing.**

a) Variant classification and b) SNV classification in PDTO models. c) Detected *TP53* mutations. d) Comparison of total detected mutations in *TP53* mut and wt PDTOs. e) Principal component analysis of RNA sequencing data from organoids shows different clusters (*TP53* mut; HPV+ and neither). Note that the two *TP53* alleles (A138V and R248Q) cluster separately.

**
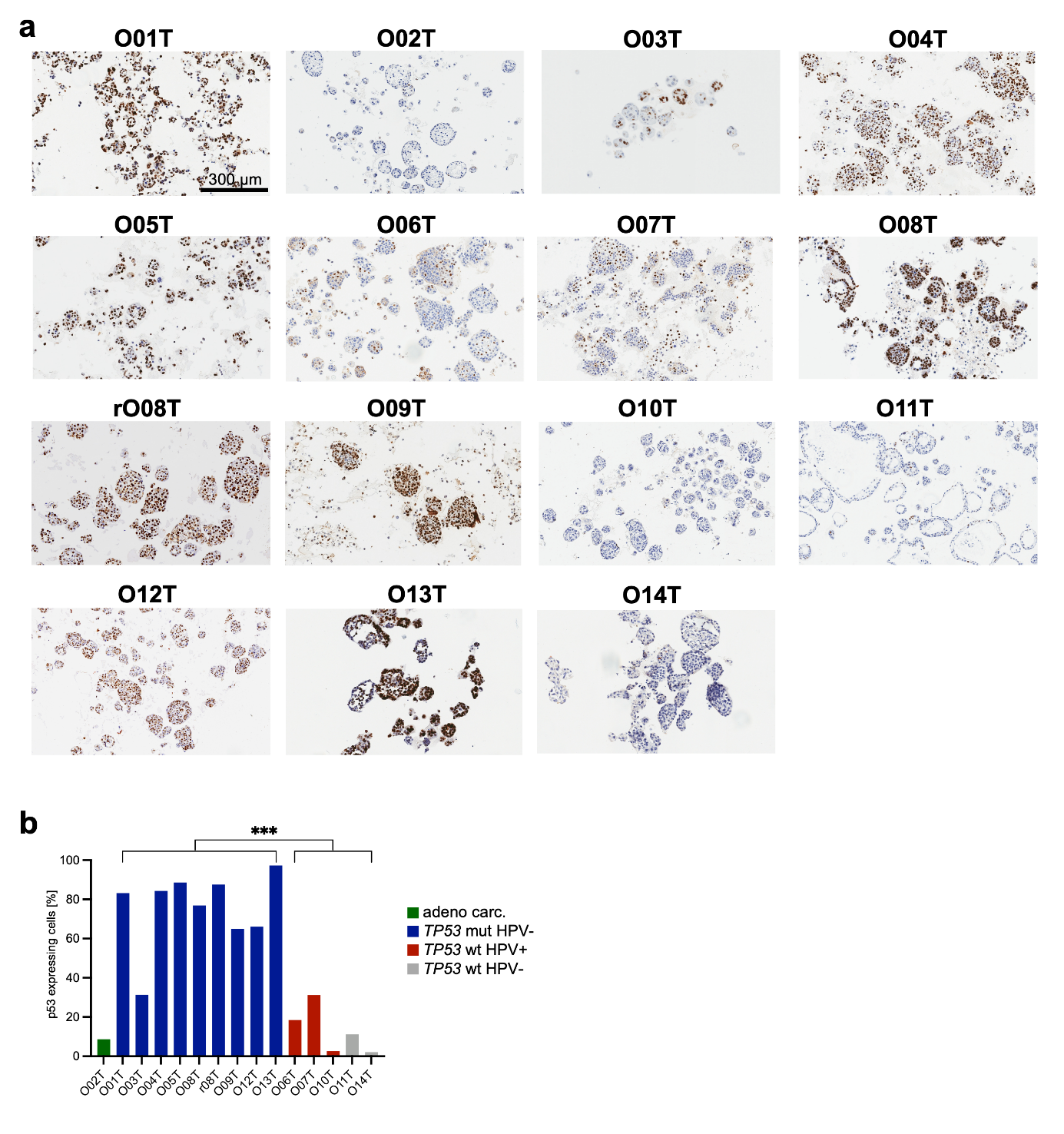
**

**Fig. S4｜p53 immunohistochemistry of HNSCC organoids.**

a) Representative immunohistochemistry images of p53 staining of PDTOs 7 days after seeding single cells. Images were captured with a 20× objective. Scalebar is 300 µm. b) Quantification of p53 expressing cells in % of individual PDTO models. TP53 mutant PDTOs show significant higher p53 expression compared to TP53 WT organoids.

**
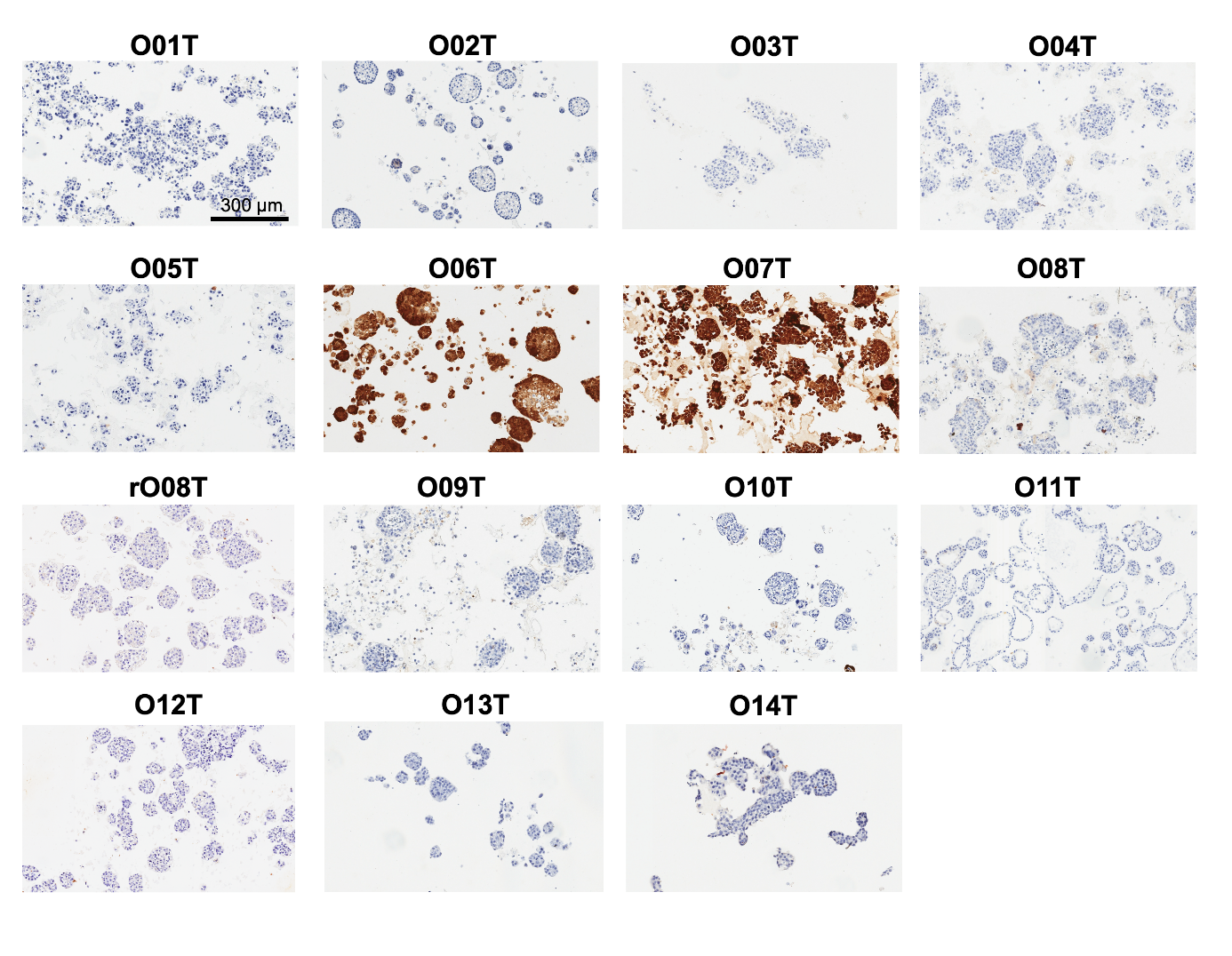
**

**Fig. S5｜p16 immunohistochemistry in HNSCC organoids as HPV surrogate marker.**

Representative immunohistochemistry images of p16 staining of PDTOs 7 days after seeding single cells. Images were captured with a 20× objective. Scalebar is 300 µm.

**
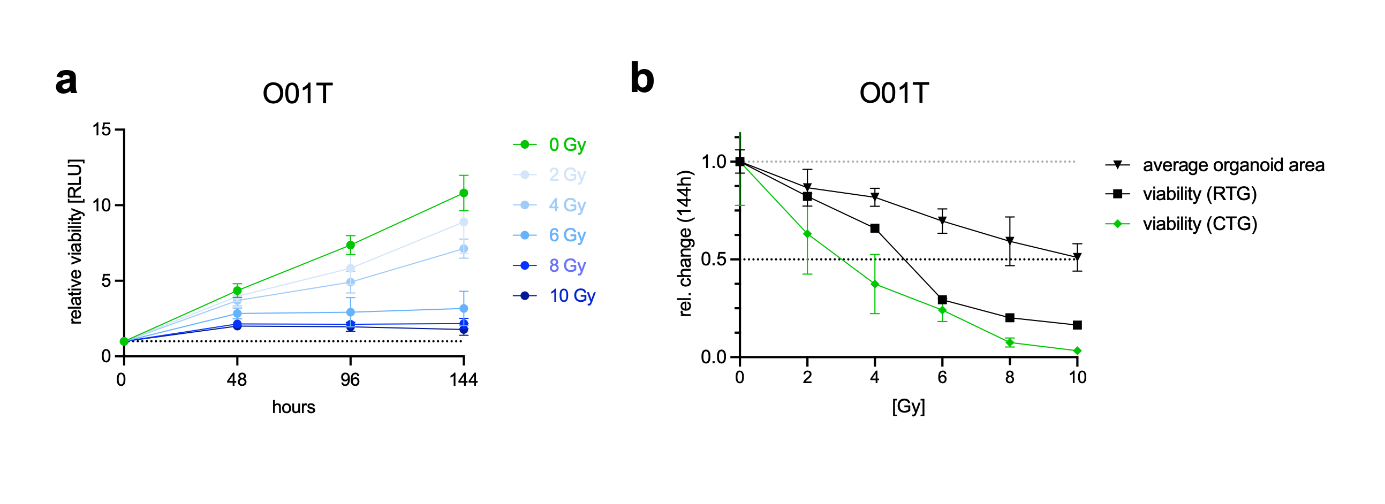
**

**Fig. S6｜Characterization and validation of the radiation response (RR) assay.**

a) Kinetic measurement of the relative cell viability using RealTime-Glo^TM^ after seeding an adjusted number of single cells and irradiation from 0 to 10 Gy two days after seeding. Measured in 6 wells each. b) Comparison of RealTime-Glo^TM^  cell viability data with CellTiter-Glo^TM^ and average organoid area was determined from morphologic images in 6 wells each.

**
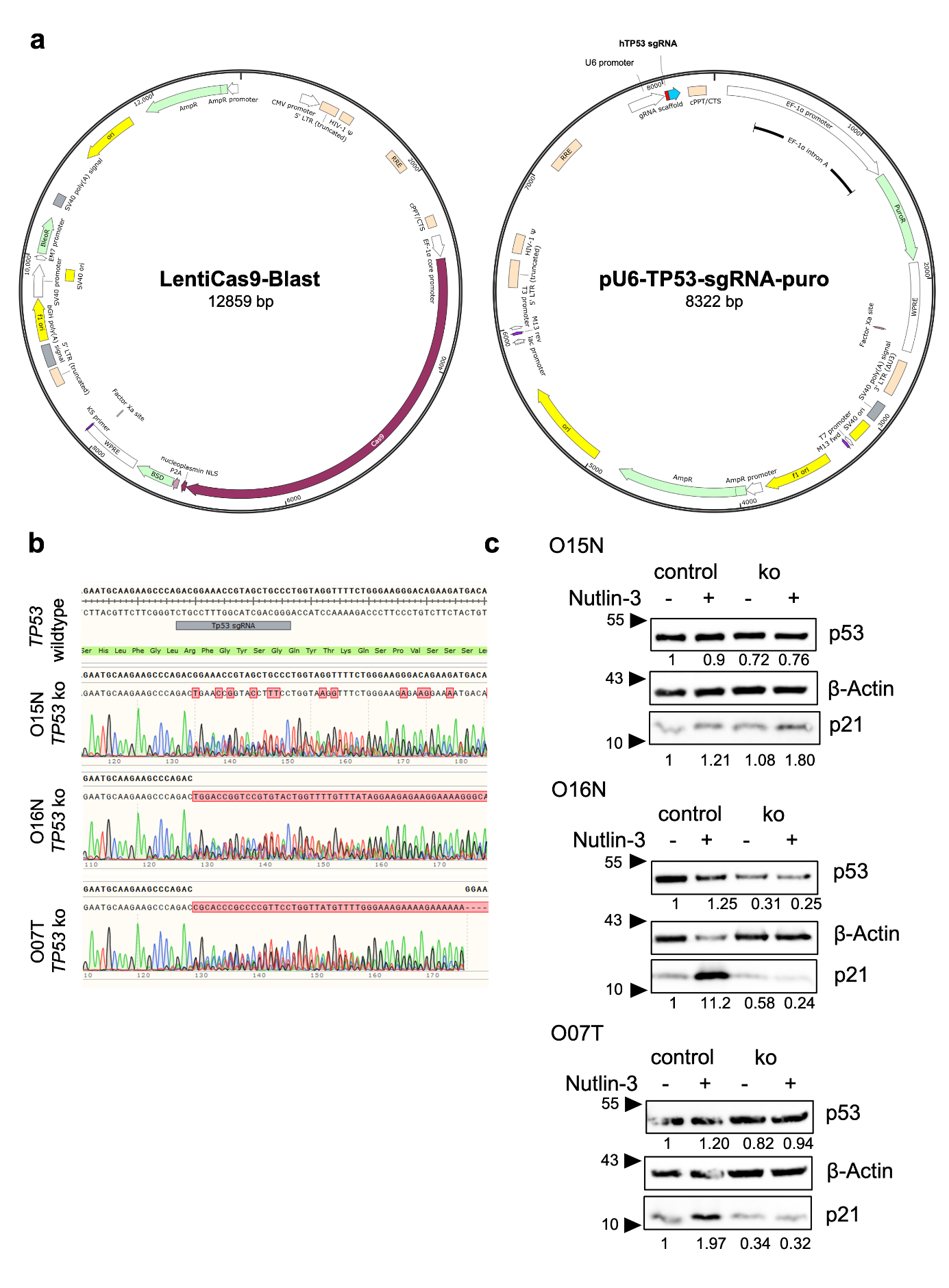
**

**Fig. S7｜Modelling the *TP53* status by CRISPR/Cas9 knock out in normal and tumor head and neck organoids.**

a) Plasmid maps for CRISPR/Cas9 knock out (ko; see methods). b) Sanger sequencing after *TP53* ko. gDNA was extracted after selection (4 passages) and amplified by PCR. Shown are aligned sequences in *TP53* WT compared to *TP53* ko in O15N, O16N and O07T 8 passages after lentiviral transduction and selection. c) Western Blot analysis of p53 and p21 in *TP53* ko and control lines. Whole cell lysates of organoids after treatment with 10 µM Nutlin-3 for 24 h. Numbers below show normalized band intensity compared to beta-Actin. Reduced p21 induction confirms a functional p53 loss.

**
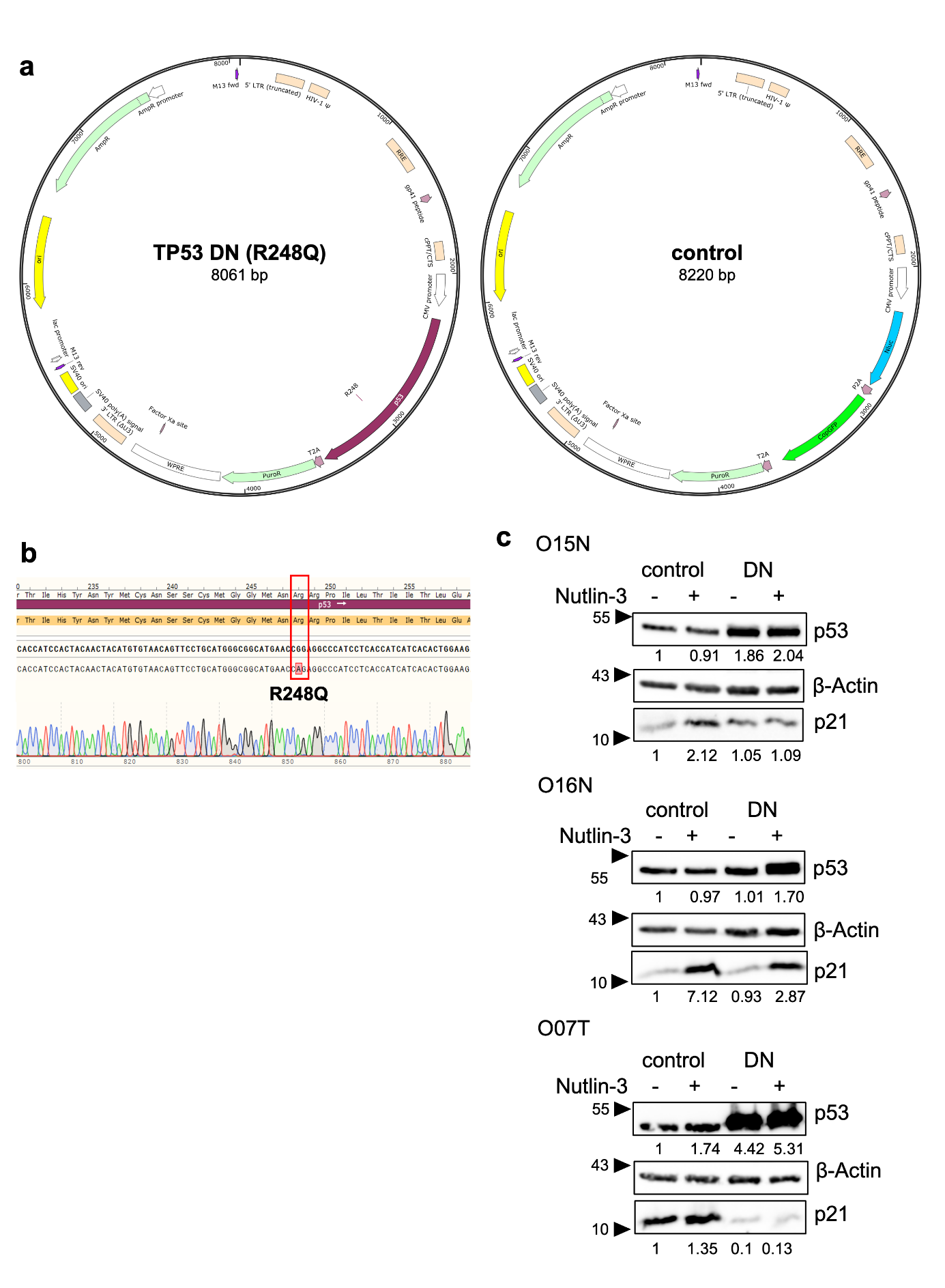
**

**Fig. S8｜Lentiviral expression of a dominant negative *TP53* variant in normal and tumor head and neck organoids.**

a) Plasmid maps (see methods). b) Sanger sequencing of the R248Q mutation in the lentiviral construct. c) Western Blot analysis of p53/p21 in *TP53* DN and control lines. Whole cell lysates of organoids after treatment with 10 µM Nutlin-3 for 24 h. Numbers below show normalized band intensity compared to beta-Actin. p21 induction in control but not *TP53* deficient lines confirms a functional p53 loss.

**
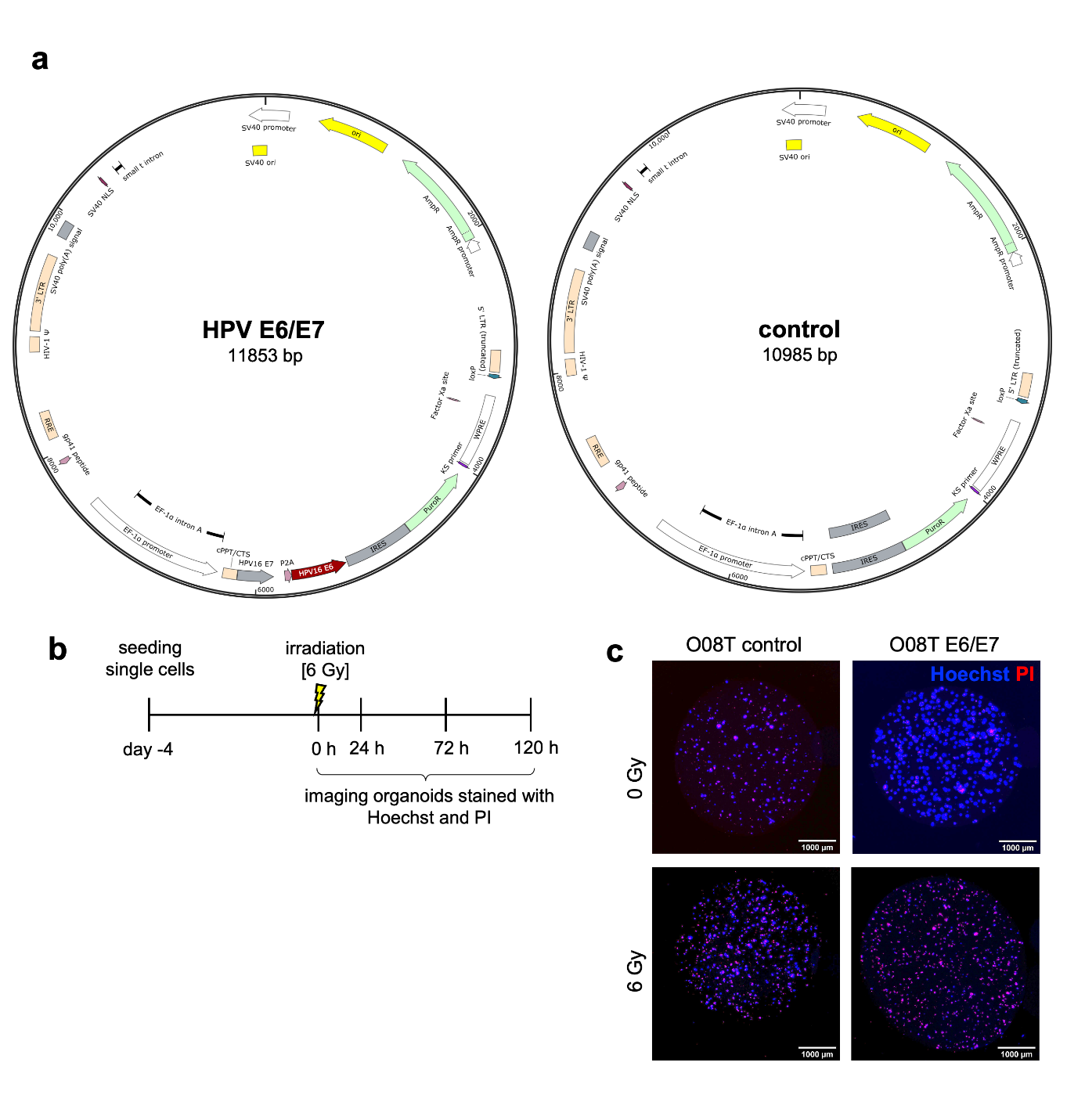
**

**Fig. S9｜ Modelling HPV status by E6/E7 overexpression in normal and tumor head and neck organoids.**

a) Plasmid maps (see methods). b) Scheme of image-based live/dead cell assay. Adjusted numbers of single cells were seeded and irradiated with 6 Gy on day 4. Organoids were incubated with Hoechst and PI 0 h, 24 h, 72 h and 120 h after the irradiation, followed by image-based analysis. c) Representative fluorescent microscopic image of O08T E6/E7 and control 120 hours after irradiation (0 or 6 Gy). Scalebars are 1000 µm.

**
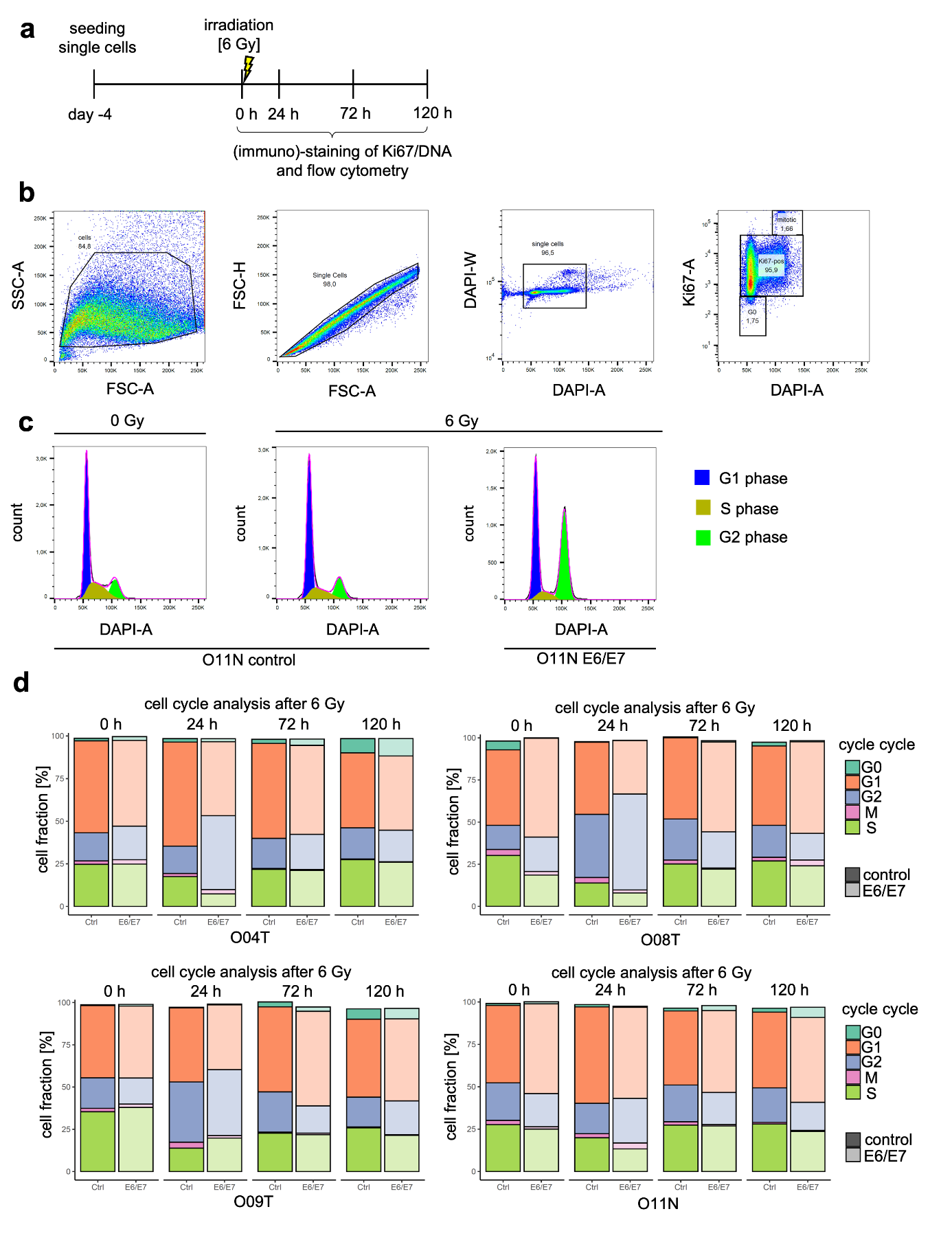
**

**Fig. S10 ｜ Cell cycle analysis after E6/E7 overexpression in normal and tumor head and neck organoids.**

a) Scheme of flow cytometry-based cell cycle assay. Adjusted single cells were seeded in parallel (for imaging and FACS), irradiated with 6 Gy, and analyzed at indicated time points. b) Gating strategy for cell cycle analysis (shown is O11N control at 0 h). c) Representative FACS data of O11N E6/E7 and O11N control 24 hours after irradiation with 6 Gy. d) Summary of flow cytometry-based cell cycle data. Cell fractions are shown in controls and upon E6/E7 overexpression.

**
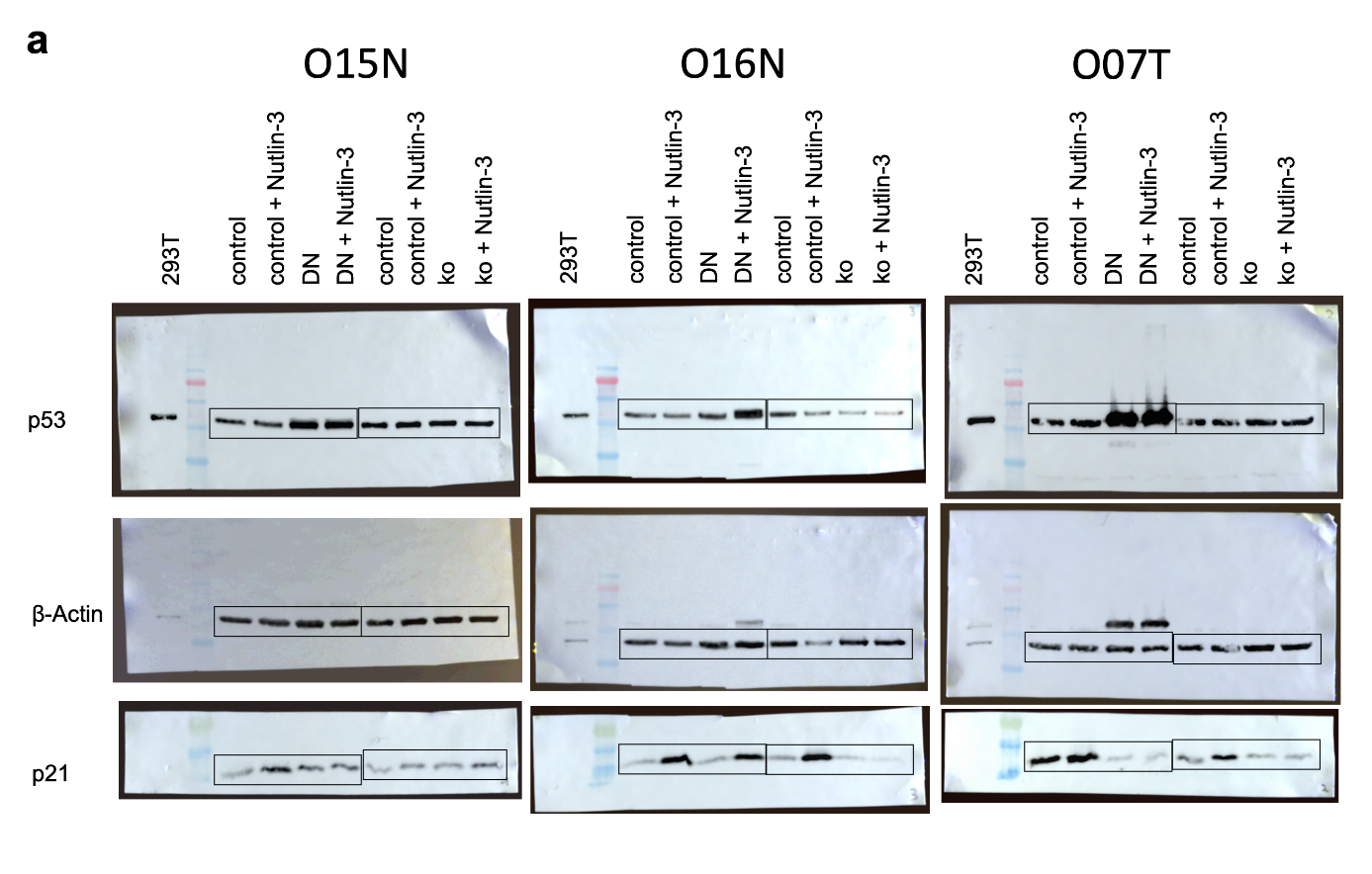
**

**Fig. S11 ｜ Raw data of Western Blot analysis in *TP53* ko, DN and control organoids.**

Scans of Western Blots shown in Supplemental Figs. S7 and S8 using antibodies against p53, p21 and beta-Actin. Boxes show the relevant regions. HEK 293T cell lysates are shown as control.
